# Nanoporous Cubic Silicon Carbide Photoanodes for Enhanced Solar Water Splitting

**DOI:** 10.1021/acsnano.1c00256

**Published:** 2021-02-19

**Authors:** Jing-Xin Jian, Valdas Jokubavicius, Mikael Syväjärvi, Rositsa Yakimova, Jianwu Sun

**Affiliations:** Department of Physics, Chemistry and Biology (IFM), Linköping University, SE-58183 Linköping, Sweden

**Keywords:** nanoporous cubic silicon carbide (3C-SiC), photoelectrochemical water splitting, solar-to-hydrogen conversion, anodization, charge-separation efficiency

## Abstract

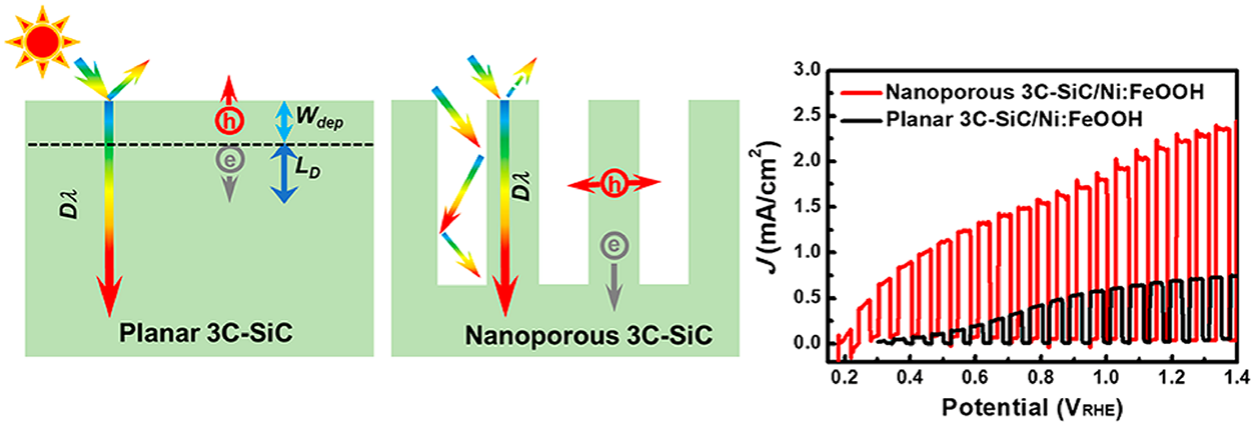

Cubic silicon carbide (3C-SiC) is a promising photoelectrode material for solar water splitting due to its relatively small band gap (2.36 eV) and its ideal energy band positions that straddle the water redox potentials. However, despite various coupled oxygen-evolution-reaction (OER) cocatalysts, it commonly exhibits a much smaller photocurrent (<∼1 mA cm^–2^) than the expected value (8 mA cm^–2^) from its band gap under AM1.5G 100 mW cm^–2^ illumination. Here, we show that a short carrier diffusion length with respect to the large light penetration depth in 3C-SiC significantly limits the charge separation, thus resulting in a small photocurrent. To overcome this drawback, this work demonstrates a facile anodization method to fabricate nanoporous 3C-SiC photoanodes coupled with Ni:FeOOH cocatalyst that evidently improve the solar water splitting performance. The optimized nanoporous 3C-SiC shows a high photocurrent density of 2.30 mA cm^–2^ at 1.23 V *versus* reversible hydrogen electrode (V_RHE_) under AM1.5G 100 mW cm^–2^ illumination, which is 3.3 times higher than that of its planar counterpart (0.69 mA cm^–2^ at 1.23 V_RHE_). We further demonstrate that the optimized nanoporous photoanode exhibits an enhanced light-harvesting efficiency (LHE) of over 93%, a high charge-separation efficiency (Φ_sep_) of 38%, and a high charge-injection efficiency (Φ_ox_) of 91% for water oxidation at 1.23 V_RHE_, which are significantly outperforming those its planar counterpart (LHE = 78%, Φ_sep_ = 28%, and Φ_ox_ = 53% at 1.23 V_RHE_). All of these properties of nanoporous 3C-SiC enable a synergetic enhancement of solar water splitting performance. This work also brings insights into the design of other indirect band gap semiconductors for solar energy conversion.

## Introduction

Photoelectrochemical (PEC) water splitting is a promising approach to convert the intermittent solar radiation into a renewable, storable, and clean chemical energy in the form of hydrogen (H_2_).^1−9^ To accomplish an efficient solar-to-hydrogen conversion in the PEC cell, the semiconductor photoelectrodes should meet certain criteria: (i) moderate band gap that can efficiently absorb visible sunlight to generate electrons and holes with enough energy to overcome the energetic barrier of water splitting, (ii) ideal band positions that straddle the water redox potentials, (iii) efficient carrier separation and transport before recombination, (iv) high activity for water splitting with low overpotential, and (v) long-term stability against corrosion in aqueous electrolytes.^10−12^ To date, there is no cost-effective single material which satisfies all of these requirements for solar water splitting.^6,10−12^ Most of the extensively studied materials such as Si, TiO_2_, Fe_2_O_3_, BiVO_4_, WO_3_, ZnO, II–VI, and III–V semiconductors exhibit either a too large band gap to harvest visible sunlight (*e.g.*, TiO_2_, ZnO, and so on) or unmatched band positions that are not able to oxidize or reduce water (*e.g.*, Si, Fe_2_O_3_, BiVO_4_, and WO_3_, *etc.*), or a poor stability in the electrolyte.^13−18^ In this regard, cubic silicon carbide (3C-SiC) has a relatively small band gap of 2.36 eV, which is close to the hypothetical ideal band gap (2.03 eV) of a single material for a maximum of the solar water splitting efficiency.^19^ Most importantly, the conduction and valence band positions of 3C-SiC ideally straddle the water redox potentials, indicating that the photogenerated carriers have enough energy to overcome the energetic barrier of water splitting without applying any external bias.^20−22^

However, 3C-SiC, as a photoelectrode material, has not been well studied due to a lack of high-quality materials.^22^ Recently, Kato and co-workers reported that a photocathode fabricated using the p-type 3C-SiC epilayer grown on 4H-SiC by chemical vapor deposition and coated with Pt nanoparticles exhibited a promising water reduction performance, which achieved a solar-to-hydrogen (STH) conversion efficiency of 0.52%.^23^ They further demonstrated that the 3C-SiC p–n junction photocathode coated with Pt showed the STH efficiency of 0.72%.^24^ However, it is still quite challenging to employ n-type 3C-SiC as a photoanode for the PEC water oxidation, which involves a four-electron process with higher energy barriers than the two-electron water reduction reaction and thus is regarded as the bottleneck for the PEC water splitting. Like most of the other semiconductor photoanodes, n-type 3C-SiC suffers from photocorrosion (surface oxidation).^20,21^ Therefore, a protective layer on 3C-SiC photoanodes is always required. Song *et al.* reported that a coating of Pt nanoparticles on the n-type 3C-SiC photoanode improved its PEC performance and protected the surface against photocorrosion.^21^

Recently, our group has demonstrated that high-quality n-type 3C-SiC films can be grown by the sublimation technique.^25−27^ By integrating efficient oxygen-evolution-reaction (OER) cocatalysts on 3C-SiC, we showed an enhanced water oxidation performance and improved stability of the 3C-SiC photoanodes.^28−30^ However, we found that despite the coating of different OER cocatalysts such as nickel oxide,^28^ iron oxyhydroxide (FeOOH),^29,30^ and nickel–iron oxyhydroxide (Ni:FeOOH) on 3C-SiC, ^29^ the photocurrent density (*J*_ph_) was limited by a maximum value of ∼1.1 mA cm^–2^ at 1.23 V *versus* reversible hydrogen electrode (V_RHE_) under AM1.5G 100 mW cm^–2^ illumination (Supporting Information Table S1), which is still far below the maximum theoretical *J*_ph_ value of 8.0 mA cm^–2^. Given the fact that 3C-SiC is an indirect band gap material, we believe one of the major factors limiting *J*_ph_ is a relatively small absorption coefficient of 3C-SiC. This would result in a large light penetration depth compared to the carrier diffusion length and space charge width. As a result, most of the photogenerated electron–hole pairs are distributed in the neutral region and recombine there, thus limiting the photon-conversion efficiency. Another factor is that the smooth surface of the as-grown planar 3C-SiC also reflects part of the sunlight, which limits the light-harvesting efficiency. To overcome these drawbacks, we propose to fabricate the nanoporous 3C-SiC photoanode for improving its PEC water splitting performance. During the past decades, it has been extensively demonstrated that a large variety of nanostructured or porous-structured semiconductor photoelectrodes such as Si, Fe_2_O_3_, BiVO_4_, and ZnO, *etc.*, could efficiently promote light harvesting, facilitate charge diffusion, and increase the active surface area, thus significantly enhancing the PEC water splitting performance.^7,12,16,18^ Although 3C-SiC exhibits promising properties for solar water splitting due to its relatively small band gap (2.36 eV) and its ideal energy band positions that straddle the water redox potentials, the fabrication of nanoporous 3C-SiC as an efficient photoelectrode for solar water splitting remains unexplored.

In this work, we showed a facile anodization method to fabricate nanoporous 3C-SiC(111) and 3C-SiC(001) photoanodes. In both cases, we demonstrated that, with coating of Ni:FeOOH as the OER cocatalyst and the protection layer, the resulting nanoporous photoanodes significantly enhanced light-harvesting efficiency, charge-separation efficiency, and charge-injection efficiency for water oxidation, thus evidently improving the overall PEC water splitting performance. The optimized nanoporous 3C-SiC(111) photoanode achieved a high photocurrent density of 2.30 mA cm^–2^ at 1.23 V_RHE_ under AM1.5G 100 mW cm^–2^ illumination, which significantly outperforms its planar counterpart (0.69 mA cm^–2^ at 1.23 V_RHE_). To our knowledge, such PEC water splitting photocurrent is the highest value ever reported for 3C-SiC as a photoanode under AM1.5G 100 mW cm^–2^ illumination (Table S1).

## Results and Discussion

### Drawback of Planar 3C-SiC Photoanodes

**Figure 1:**
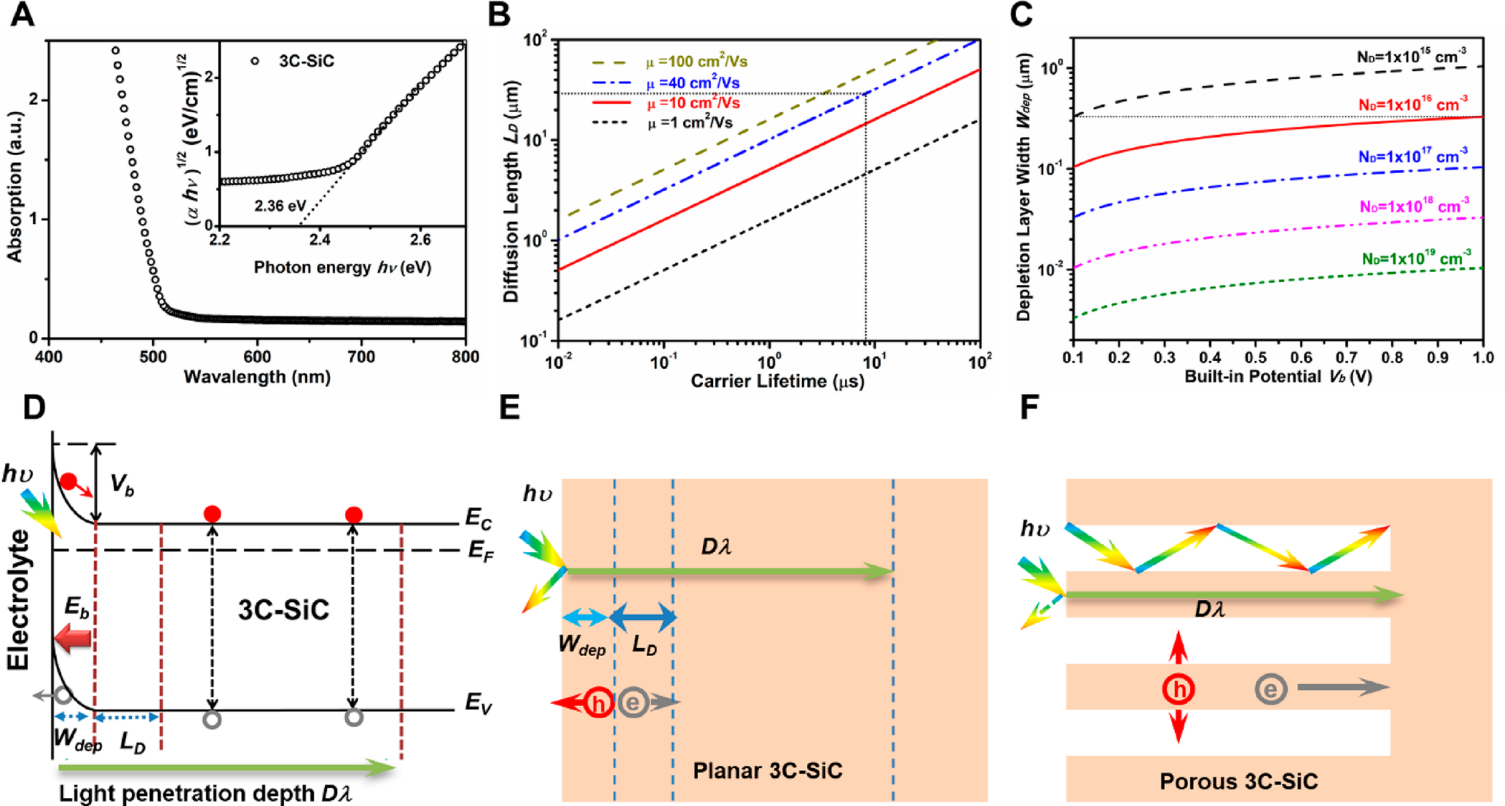
Drawback of planar 3C-SiC photoanodes. (A) Absorption spectrum and Tauc plot (inset) of high-quality 3C-SiC(111). (B) calculated diffusion length (*L*_D_) as a function of the carrier lifetime (τ) with different hole mobilities (μ). (C) calculated width of space charge region (*W*_dep_) as a function of the built-in potentials (*V*_b_) with different doping concentrations (*N*_D_). (D) Schematic illustration of *W*_dep_, *L*_D_, and light penetration depth (*D*_λ_) in 3C-SiC photoanode. (E, F) schematic comparison of charge separation and diffusion in planar 3C-SiC and nanoporous 3C-SiC photoanode.

Figure 1A shows the absorption spectrum of a 300 μm thick freestanding 3C-SiC(111) sample grown by sublimation epitaxy,^26,27^ which exhibits a sharp band-edge absorption. The Tauc plot shown in the inset of Figure 1A yields an optical band gap of 2.36 eV, which is consistent with the reported band gap of 3C-SiC.^31^ Due to the nature of an indirect band gap semiconductor, 3C-SiC exhibits a smaller absorption coefficient (α) than direct band gap materials. Patrick and Choyke reported that the absorption coefficient of the n-type 3C-SiC crystal is ∼63 cm^–1^ at 2.375 eV, giving rise to a rather large light penetration depth (*D*_λ_ = 1/α) of 159 μm at 2.375 eV (522 nm).^32^ Solangi and Chaudhry reported that the absorption coefficient of the n-type 3C-SiC epilayer (doping density is 5 × 10^16^ cm^–3^) grown on Si is around ∼100 cm^–1^ at 2.4 eV, which gives *D*_λ_ ∼ 100 μm at 2.4 eV.^33^ These results indicate that at least a 100 μm thick 3C-SiC layer is required to fully absorb the sunlight with photon energies larger than its band gap. However, only the photogenerated electrons and holes in the space charge region and the diffusion length region can be separated by the built-in electric field (*E*_b_) and thus be harvested for PEC water splitting. For the photoanode, the carrier diffusion length (*L*_D_) and the width of space charge region (*W*_dep_) are given by
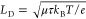
1
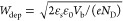
2where μ is the hole mobility, τ is the hole lifetime, *k*_B_*T*/*e* is the product of the Boltzmann constant and the temperature divided by the elementary charge, ε_s_ is the dielectric constant of 3C-SiC, ε_0_ is the vacuum permittivity, *V*_b_ is the built-in potential, and *N*_D_ is the donor concentration.

We calculated *L*_D_ of 3C-SiC as a function of hole lifetimes (Figure 1B). The reported hole mobility in 3C-SiC ranges from 1 to 40 cm^2^ V^–1^ s^–1^.^34,35^ Due the presence of structural defects, 3C-SiC commonly exhibits rather short hole lifetimes from a few to 120 ns.^36^ Recently, we have demonstrated that high-quality 3C-SiC grown by sublimation epitaxy exhibits a long hole lifetime of 8.2 μs.^25^ With the reported highest hole mobility (40 cm^2^ V^–1^ s^–1^) and the lifetime (8.2 μs), the maximum value of *L*_D_ is calculated to be 29.1 μm (Figure 1B). Due to a background nitrogen doping, the typical donor concentration in 3C-SiC is around ∼1 × 10^16^ cm^–3^,^25^ giving rise to a *W*_dep_ of 0.3 μm with an assumption of the *V*_b_ as high as 1 V (Figure 1C). Therefore, the maximum value of *W*_dep_ + *L*_D_ is estimated to be 29.4 μm, which is much smaller than the light penetration depth of ∼100 μm at 2.4 eV. This indicates that most photogenerated carriers in 3C-SiC are distributed within the neutral region and recombine there (Figure 1D,E), thus resulting in a relatively small photocurrent. To overcome this drawback of the planar photoanode, nanoporous 3C-SiC is proposed in this work. As shown in Figure 1F, independent of the large light penetration depth, the nanoporous 3C-SiC photoanode would shorten the distance for the hole transfer and enable hole transfer through different directions, thus improving the charge-separation efficiency. Moreover, the nanoporous structure would also reduce light reflection due to a light trapping effect and provide a large active surface for PEC reaction. These properties are expected to significantly increase the PEC water splitting efficiency.

### Nanoporous 3C-SiC(111) Photoanodes

**Figure 2:**
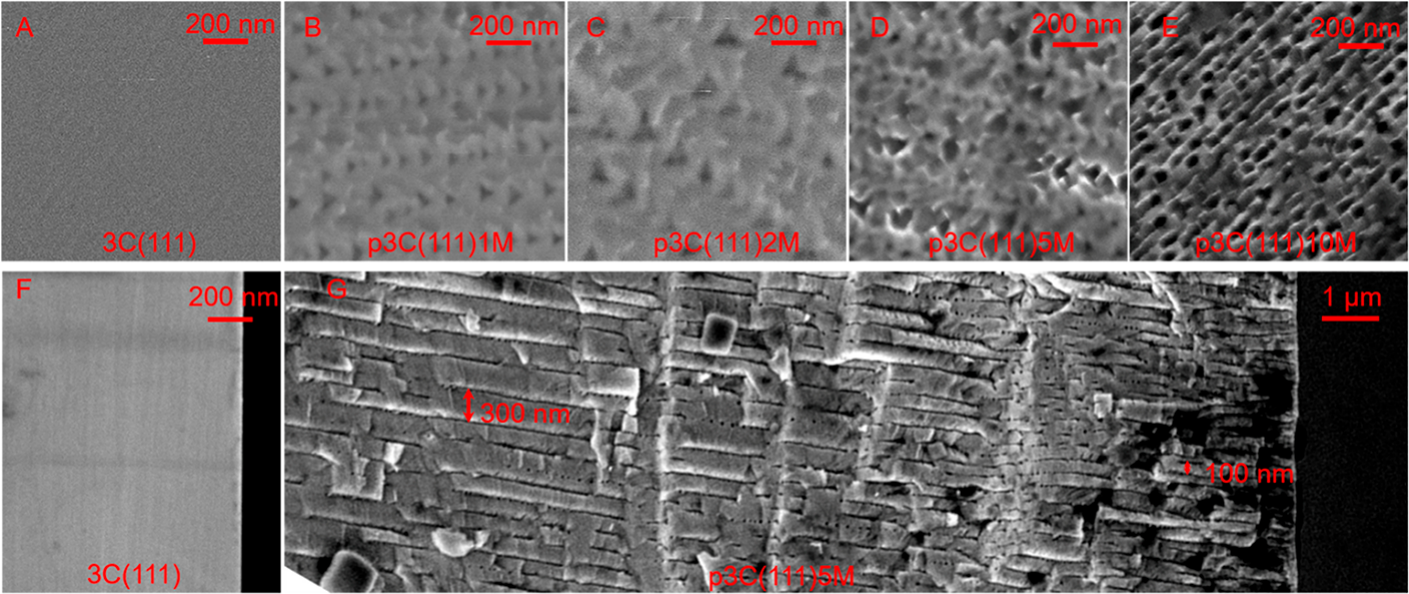
Morphologies of nanoporous 3C-SiC(111). Top-view SEM images of the planar 3C(111) (A) and nanoporous p3C(111)*x*M, where *x*M represents the anodization times of 1, 2, 5, and 10 min, respectively (B–E). Comparison of the cross-sectional SEM images of the planar 3C(111) (F) and nanoporous p3C(111)5M (G).

To demonstrate the advantages of the proposed nanoporous 3C-SiC for solar water splitting, we prepared nanoporous 3C-SiC(111) by a facile anodization method as described in Methods. The resulting samples are denoted as p3C(111)*x*M, where *x*M represents the anodization times of 1, 2, 5, and 10 min, respectively. The scanning electron microscopy (SEM) images shown in Figure 2 compare morphologies of planar 3C(111) and nanoporous p3C(111)*x*M. With increasing the anodization time from 1 to 5 min, the parallelly arranged triangle-shaped holes were formed and enlarged on the surface of 3C-SiC(111). The formation of triangle-shaped holes is related to the ⟨111⟩ orientation. However, after 10 min anodization, the sample showed very dense interconnected holes and the triangle shape disappeared. This is probably due to the severe etching. The cross-sectional SEM images show the prolongation of anodization time increases the depth of the porous layer, which is about 5.6, 12.6, 24.1, and 25.4 μm after anodization of 1, 2, 5, 10 min, respectively (Figure S1). A high magnification of the cross-sectional SEM of p3C(111)5M shows the columnar pore structure, with the diameter of columns gradually increasing from 100 nm near the surface to 300 nm inside the material (Figure 1G). However, the sample with 10 min anodization exhibits very large voids in the outermost ∼10 μm thick layer (Figure S1D), indicating the deterioration of the crystalline quality due to severe anodization etching.

To compare planar and nanoporous 3C(111) photoanodes and protect them against photocorrosion, we deposited Ni:FeOOH as the OER cocatalyst on these photoanodes at the same time. The prepared planar and nanoporous photoanodes are denoted as 3C(111)/NiFe and p3C(111)*x*M/NiFe, respectively. The Ni:FeOOH, as a cheap and earth-abundant material, has been widely used as an efficient and stable OER cocatalyst for PEC water splitting, especially in alkaline conditions.^29,37,38^ As seen in Figure S2, the Ni:FeOOH on planar 3C(111) is composed of a network-like layer with a thickness of ∼140 nm. In contrast, on nanoporous p3C(111)5M/NiFe, Ni:FeOOH consists of a 160 nm thick layer of nanoparticles. This morphology difference is probably due to the size confinement of the Ni:FeOOH nucleation in the nanoporous structure. The cross-sectional SEM image of p3C(111)5M/NiFe and its energy-dispersive X-ray spectroscopy (EDXS) and the elemental mapping measurements confirm that Ni:FeOOH is also deposited inside of the pore structures (Figure S2I–M). As seen in Figure S2H,M, the EDXS spectra collected on the surface and the pore structure of p3C(111)5M/NiFe show similar Ni, Fe, and O signals. Meanwhile, the ratio of Ni and Fe elements detected by EDXS analysis is around 8:100, which is close to the reported optimal ratio of Ni:FeOOH cocatalyst.^37^ The X-ray diffraction (XRD) patterns of 3C(111)/NiFe and p3C(111)5M/NiFe display identical diffraction peaks, corresponding to the β-FeOOH phases (Figure S3).^37,39^

**Figure 3:**
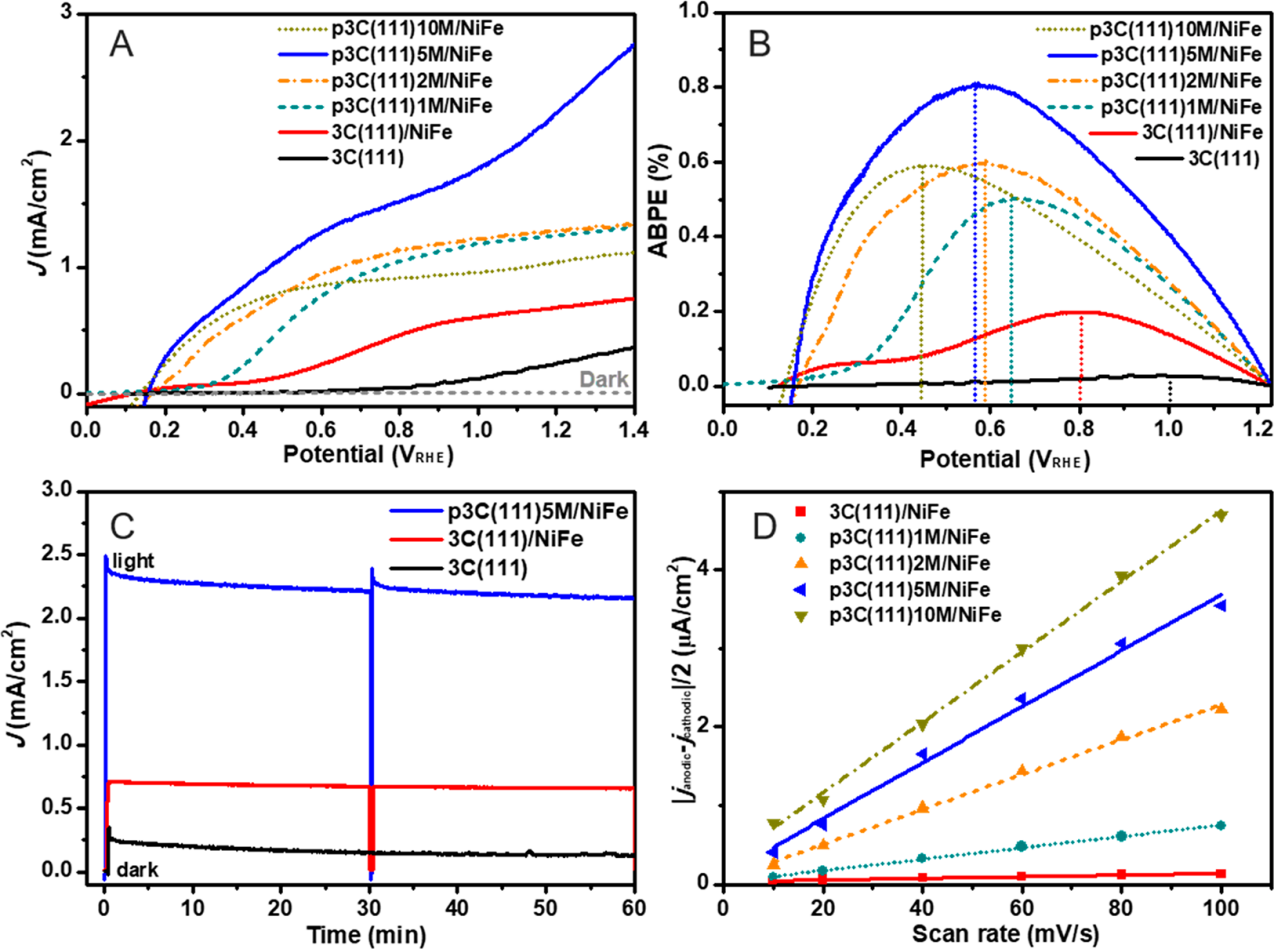
PEC water oxidation performance of planar and nanoporous 3C-SiC(111) photoanodes. Current density–potential (*J*–*V*) curves (A) and ABPE (B) of planar 3C(111), planar 3C(111)/NiFe, and nanoporous p3C(111)*x*M/NiFe photoanodes, where *x*M represents the anodization times of 1, 2, 5, and 10 min, respectively. (C) Photocurrent density–time (*J*–*t*) curves of 3C(111), 3C(111)/NiFe, and p3C(111)5M/NiFe photoanodes at 1.23 V_RHE_ under illumination. All of the PEC measurements were carried out in 1.0 M NaOH electrolyte under AM1.5G 100 mW cm^–2^ illumination. (D) Plots of (|*j*_anodic_–*j*_cathodic_|/2) at 0.55 V_RHE_ as a function of the scan rate, showing the extraction of double-layer capacitance (*C*_dl_) for 3C(111)/NiFe and p3C(111)*x*M/NiFe photoanodes. The lines show the linear fitting plots, whose slopes correspond to *C*_dl_ according to the equation *C*_dl_ = *I*/(d*v*/d*t*).

The PEC water splitting performances of the planar 3C(111), 3C(111)/NiFe, and nanoporous p3C(111)*x*M/NiFe photoanodes were measured in 1.0 M NaOH electrolyte under AM1.5G 100 mW cm^–2^ illumination. As seen in Figures 3A and S4 (*J*–*V* curves under chopped 1 sun illumination), with a coating of Ni:FeOOH cocatalyst, the 3C(111)/NiFe photoanode significantly reduced the onset potential (*E*_onset_) and enhanced the photocurrent density (*J*_ph_) compared to pristine 3C-SiC(111), indicating that Ni:FeOOH boosts the OER activity. As proposed, the nanoporous 3C(111)*x*M/NiFe photoanodes dramatically enhanced the photocurrent density compared to the planar counterpart (Figures 3A and S4; Table 1). With increasing the anodization times from 1 to 5 min, the resulting photoanodes exhibited an increased photocurrent due to the increase of the porous layer depth. However, the photoanode with 10 min anodization showed a decreased photocurrent. This is probably caused by the deterioration of the outermost 3C-SiC(111) due to severe anodization etching (Figure S1D). The p3C(111)5M/NiFe photoanode achieved the highest *J*_ph_ of 2.30 mA cm^–2^ at 1.23 V_RHE_, which is 3.3 times higher than that of the planar 3C(111)/NiFe and the highest photocurrent among the reported values of 3C-SiC photoanodes (see Table S1).

**Table 1:** PEC Water Splitting Performance of Planar 3C(111), Planar 3C(111)/NiFe, and Nanoporous p3C(111)*x*M/NiFe Photoanodes, Where *x*M Represents the Anodization Times of 1, 2, 5, and 10 min, Respectively^a^

sample	*J* at 1.23 V_RHE_ (mA cm^–2^)	*E*_onset_ (V_RHE_)	max ABPE (%)	*E*_max_ at ABPE_max_ (V_RHE_)	ff (%)	*C*_dl_ (μF cm^–2^)	rel surf area	LHE at 450 nm (%)	*J*_Abs_ (mA cm^–2^)
planar 3C(111)	0.25	∼0.4	0.03	1.0	9				
planar 3C(111)/NiFe	0.69	∼0.2	0.20	0.80	29	1.05	1.0	78	4.67
p3C(111)1M/NiFe	1.26	∼0.2	0.50	0.65	41	7.36	7.0	86	5.67
p3C(111)2M/NiFe	1.30	∼0.2	0.60	0.58	47	22.20	21.1	90	6.28
**p3C(111)5M/NiFe**	**2.30**	**∼0.2**	**0.81**	**0.56**	**36**	**35.70**	**34.0**	**93**	**6.67**
p3C(111)10M/NiFe	1.05	∼0.2	0.59	0.45	58	44.80	42.7	97	7.40

a The best performance is highlighted in bold.

Moreover, the nanoporous 3C-SiC photoanodes exhibited a much steeper increase of photocurrent than the planar counterpart. The p3C(111)5M/NiFe showed a precipitous photocurrent increase at as low as ∼0.2 V_RHE_, which is a cathodic shift of ∼0.3 V compared to its planar counterpart 3C(111)/NiFe. This power characteristic can be clearly demonstrated by the fill factor (ff), which is defined as ff = *J*_mp_ (1.23 – *V*_mp_)/[*J*_sc_(1.23 – *E*_onset_)], where *J*_mp_ and *V*_mp_ are the photocurrent density and potential at the maximum power point and *J*_sc_ is the photocurrent density at 1.23 V_RHE_. As shown in Figure S5 and Table 1, the fill factors of the nanoporous photoanodes were substantially increased. Moreover, the potential at the maximum power point (*V*_mp_) was decreased from ∼1.0 V_RHE_ for the planar 3C(001) and ∼0.8 V_RHE_ for the planar 3C(001)/NiFe to ∼0.6 V_RHE_ for the nanoporous 3C(001)5M/NiFe (Figure S5).

Figure 3B shows the applied bias photon-to-current efficiency (ABPE) curves for the pristine 3C(111), planar 3C(111)/NiFe, and nanoporous p3C(111)*x*M/NiFe photoanodes. ABPE is given by the following equation: ABPE = *J*_ph_(1.23 – *V*_app_)/*P*_AM1.5G_, where *P*_AM1.5G_ is the light density of simulated sunlight (AM1.5G 100 mW cm^–2^). The nanoporous photoanodes demonstrated a significant enhancement of ABPE and a reduced potential at the maximum ABPE. The p3C(111)5M/NiFe photoanode exhibited a maximum ABPE of 0.81% at a low applied potential of 0.56 V_RHE_, which is the highest photoconversion efficiency ever reported for 3C-SiC photoanodes (Table S1). In contrast, the planar 3C(111)/NiFe showed much lower ABPE values of 0.20% at a higher applied potential of 0.80 V_RHE_ (Table 1).

The photocurrent density–time (*J*–*t*) curves recorded at 1.23 V_RHE_ for the planar 3C(111), 3C(111)/NiFe, and nanoporous p3C(111)5M/NiFe photoanodes are shown in Figure 3C. Under the 1 sun illumination for 60 min, the *J*_ph_ of the planar 3C(111) photoanode decreased from 0.35 to 0.13 mA cm^–2^, indicating a 63% loss of its initial *J*_ph_. It has been reported that 3C-SiC photoanodes suffered from a photocorrosion, which is a surface oxidation reaction forming SiO_2_ (3C-SiC + 4H_2_O + 8h^+^ → SiO_2_ + CO_2_ + 8H^+^).^21^ For the p3C(111)5M/NiFe photoanode, the photocurrent retained 94% of its initial *J*_ph_ of 2.30 mA cm^–2^ after 60 min illumination. Meanwhile, the evolved O_2_ over the p3C(111)5M/NiFe photoanode was detected by gas chromatography and the Faradaic efficiency of O_2_ was determined to be 75% (Table S2).

To quantify the effective electrochemically active surface area, cyclic voltammetry measurements were employed to extract the double-layer capacitance (*C*_dl_) of the planar and nanoporous photoanodes (Figure S6), according to the reported method.^7,38,40^Figure 3D shows the plots of the half-difference of the anodic and cathodic current density (|*j*_anodic_ – *j*_cathodic_|/2) at 0.55 V_RHE_ as a function of the scan rate. The slopes of these linear plots gave rise to the geometric *C*_dl_, which is proportional to the effective surface area of the photoanode. From a comparison of the extracted *C*_dl_, we found that the electrochemically active surface area of the nanoporous photoanodes was significantly increased by increasing the anodization time, consistent with the increased depth of nanoporous layer. Notably, the surface area of p3C(111)5M/NiFe is 34 times larger than that of the planar 3C(111)/NiFe (Table 1).

### Nanoporous 3C-SiC(001) Photoanodes

**Figure 4:**
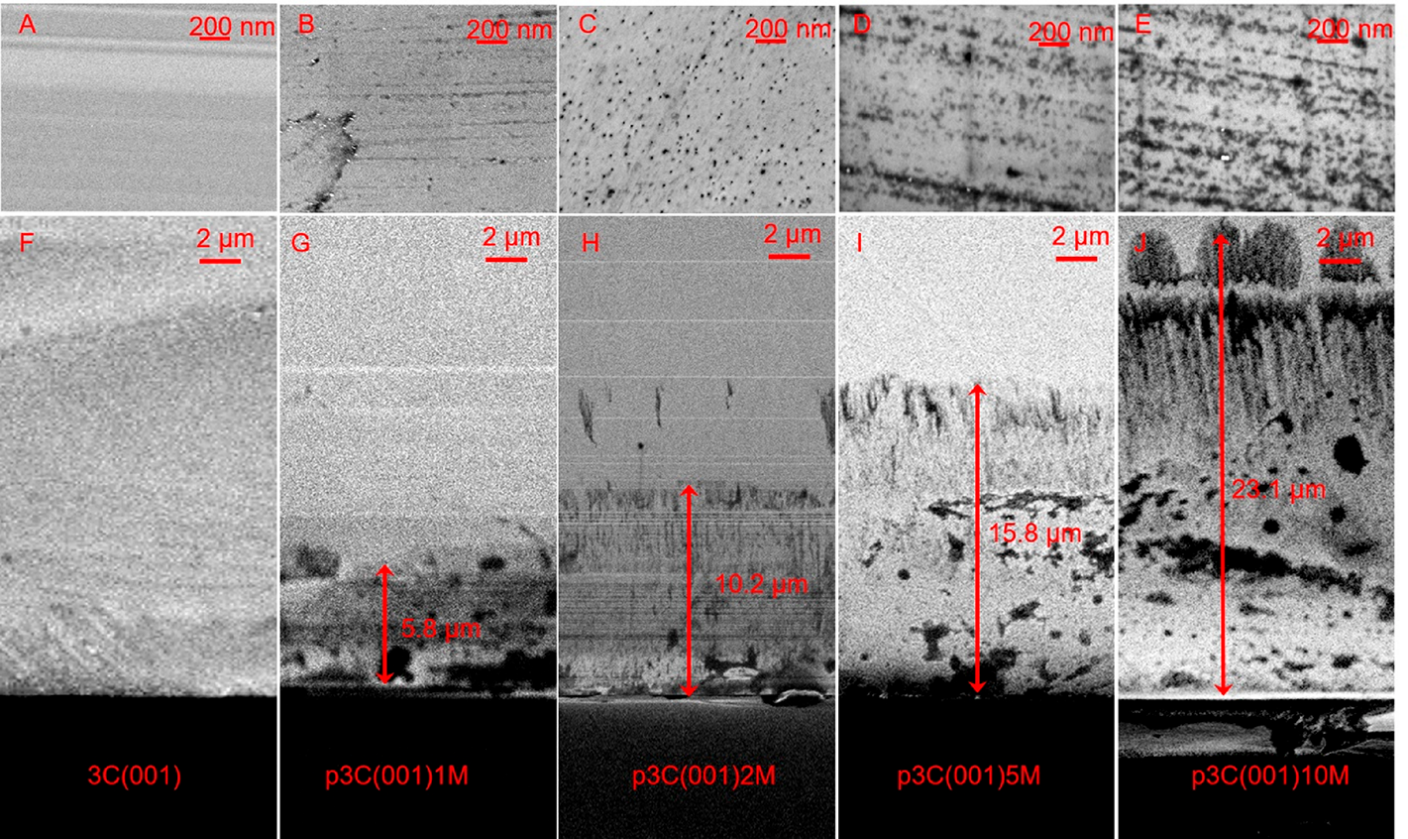
Morphologies of nanoporous 3C-SiC(001). Top view SEM images of planar 3C(001) (A) and nanoporous p3C(001)*x*M (B–E), where *x*M represents the anodization times of 1, 2, 5, and 10 min, respectively. Cross-sectional SEM images of planar 3C(001) (F) and nanoporous p3C(001)*x*M (G–J).

The nanoporous 3C-SiC(001) photoanodes were prepared by the anodization method using the same conditions as for nanoporous 3C-SiC(111). The prepared samples are denoted as p3C(001)*x*M, where *x*M represents the anodization times of 1, 2, 5, and 10 min, respectively. Figure 4 shows the morphologies of planar 3C(001) and nanoporous p3C(001)*x*M. With increasing anodization time, more densely arranged holes on the 3C(001) surface were clearly observed. Unlike 3C(111), those holes do not show any triangular shape. The different shapes of etched holes on 3C(111) and 3C(001) are related to the different crystalline orientations. As the surface of the commercial 3C(001) wafer was mechanically polished, the anodization etching also boosts the scratches as seen in Figure 4D,E. From the cross-sectional SEM images, the depths of the nanoporous layer are 5.8, 10.2, 15.8, and 23.1 μm for the samples after 1, 2, 5, and 10 min of anodization (Figure 4G–J). Similar to nanoporous 3C-SiC(111), 10 min anodization also resulted in large voids in the outermost layer, indicating the deterioration of the crystalline quality. To evaluate the PEC performance of the nanoporous 3C-SiC(001) photoanodes, we employed the same conditions as used for 3C-SiC(111) to deposit Ni:FeOOH on 3C(001) and p3C(001)*x*M photoanodes at the same time, hereby denoted as 3C(001)/NiFe and p3C(001)*x*M/NiFe, respectively. As for the planar case, the deposited Ni:FeOOH on 3C(001) exhibited the same network-like layer as on 3C(111), as seen in Figure S7A. For the nanoporous case, the Ni:FeOOH on p3C(001)5M showed a nanoparticle morphology (Figure S7G), identical to that on p3C(111)5M. In both cases, the thicknesses of Ni:FeOOH are similar to those on 3C(111). The EDXS and the elemental mapping results confirmed that Ni:FeOOH was also deposited into the pore structure of nanoporous 3C(001), as shown in Figure S7.

**Figure 5:**
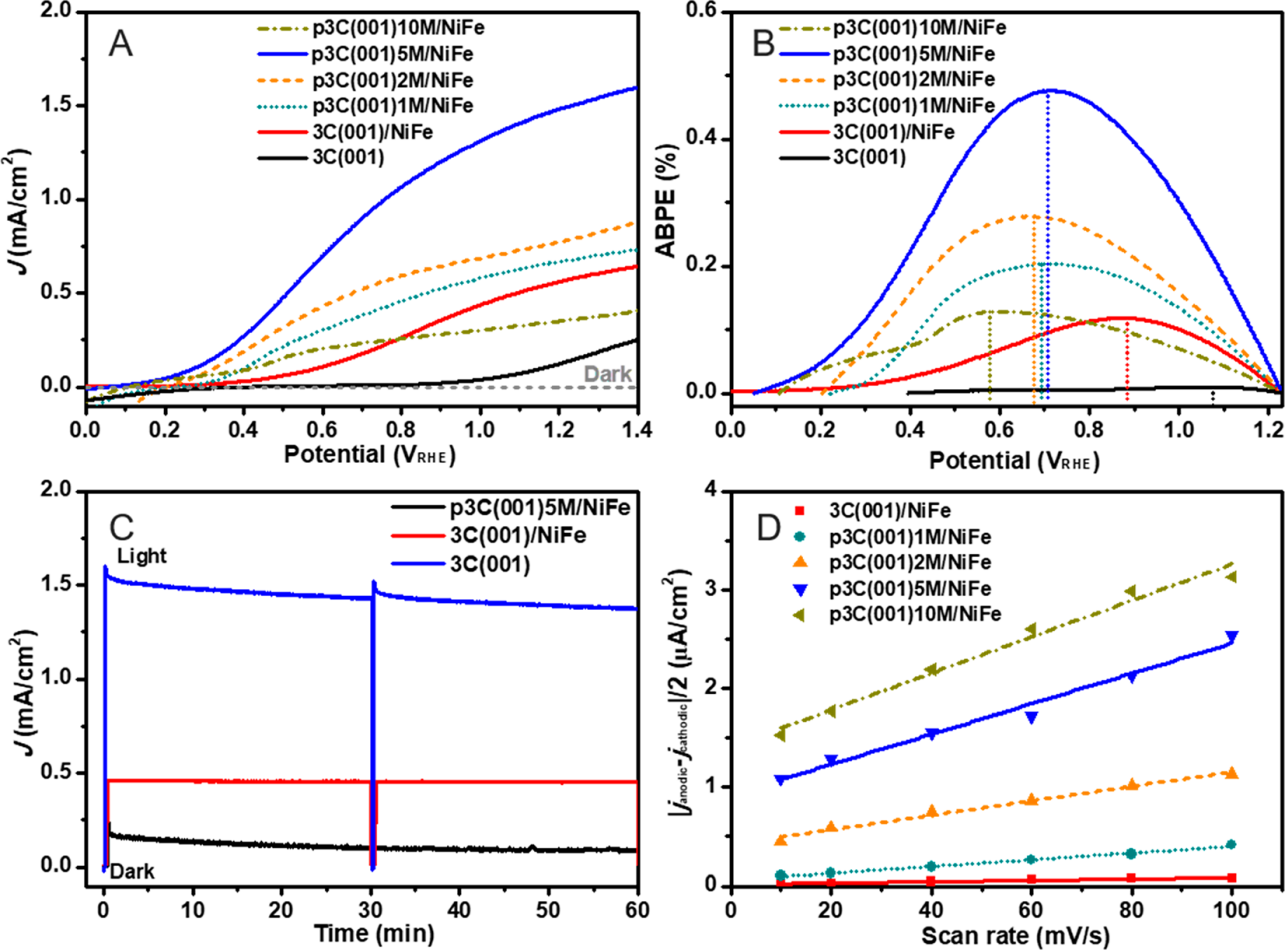
PEC water oxidation performance of planar and nanoporous 3C-SiC(001) photoanodes. *J*–*V* curves (A) and ABPE (B) of planar 3C(001), planar 3C(001)/NiFe, and nanoporous p3C(001)*x*M/NiFe photoanodes, where *x*M represents the anodization times of 1, 2, 5, and 10 min, respectively. (C) *J*–*t* curves of 3C(001), 3C(001)/NiFe and p3C(001)5M/NiFe photoanodes at 1.23 V_RHE_ under illumination. All of the PEC measurements were carried out in 1.0 M NaOH electrolyte under AM1.5G 100 mW cm^–2^ illumination. (D) Plots of (|*j*_anodic_–*j*_cathodic_|/2) at 0.55 V_RHE_ as a function of the scan rate, showing the extraction of double-layer capacitance (*C*_dl_) for 3C(001)/NiFe and p3C(001)*x*M/NiFe photoanodes. The lines show the linear fitting plots, whose slopes correspond to *C*_dl_ according to the equation *C*_dl_ = *I*/(d*v*/d*t*).

Figures 5 and S8 (*J*–*V* curves under chopped 1 sun illumination) show the PEC water splitting results of the planar 3C(001)/NiFe and nanoporous 3C(001)*x*M/NiFe photoanodes, which are quite similar to the PEC results of the corresponding 3C(111) photoanodes. The nanoporous 3C(001)*x*M/NiFe photoanodes exhibit enhanced ABPE, ff values, and surface area (Table 2 and Figures S9 and S10), but their overall PEC performance is lower than that of the nanoporous 3C(111). Among all 3C(001)*x*M/NiFe photoanodes, p3C(001)5M/NiFe gives the highest photocurrent of 1.50 mA cm^–2^ at 1.23 V_RHE_, which is 2.6 times higher than that of the planar 3C(001)/NiFe photoanode (Table 2) but still lower than the photocurrent (2.30 mA cm^–2^) of p3C(111)5M/NiFe prepared at the same conditions. Moreover, electrochemically active surface areas of p3C(001)*x*M/NiFe are respectively smaller than the corresponding p3C(111)*x*M/NiFe fabricated under the same conditions (Table 2). This result is probably due to the presence of the columnar pore structures in nanoporous p3C(111)*x*M/NiFe (Figure 2G), which might provide larger electrochemically active surface areas.

**Table 2:** PEC Water Splitting Performance of Planar 3C(001), Planar 3C(001)/NiFe, and Nanoporous p3C(001)*x*M/NiFe Photoanodes, Where *x*M Represents the Anodization Times of 1, 2, 5, and 10 min, Respectively^a^

sample	*J* at 1.23 V_RHE_ (mA cm^–2^)	*E*_onset_ (V_RHE_)	max ABPE (%)	*V* at ABPE_max_ (V_RHE_)	ff (%)	*C*_dl_ (μF cm^–2^)	rel surf area	LHE at 450 nm (%)	*J*_Abs_ (mA cm^–2^)
planar 3C(001)	0.14	∼0.4	0.01	1.07	8				
planar 3C(001)/NiFe	0.58	∼0.2	0.12	0.88	20	0.67	1.0	76	4.79
p3C(001)1M/NiFe	0.68	∼0.2	0.20	0.69	29	3.38	5.0	88	5.86
p3C(001)2M/NiFe	0.79	∼0.2	0.28	0.67	34	7.34	11.0	91	6.41
**p3C(001)5M/NiFe**	**1.50**	**∼0.2**	**0.48**	**0.71**	**31**	**15.30**	**22.8**	**96**	**7.01**
p3C(001)10M/NiFe	0.36	∼0.2	0.13	0.58	35	18.40	27.5	99	7.42

a The best performance is highlighted in bold.

### Understanding the Improvement in PEC Performance of Nanoporous 3C-SiC Photoanodes

**Figure 6:**
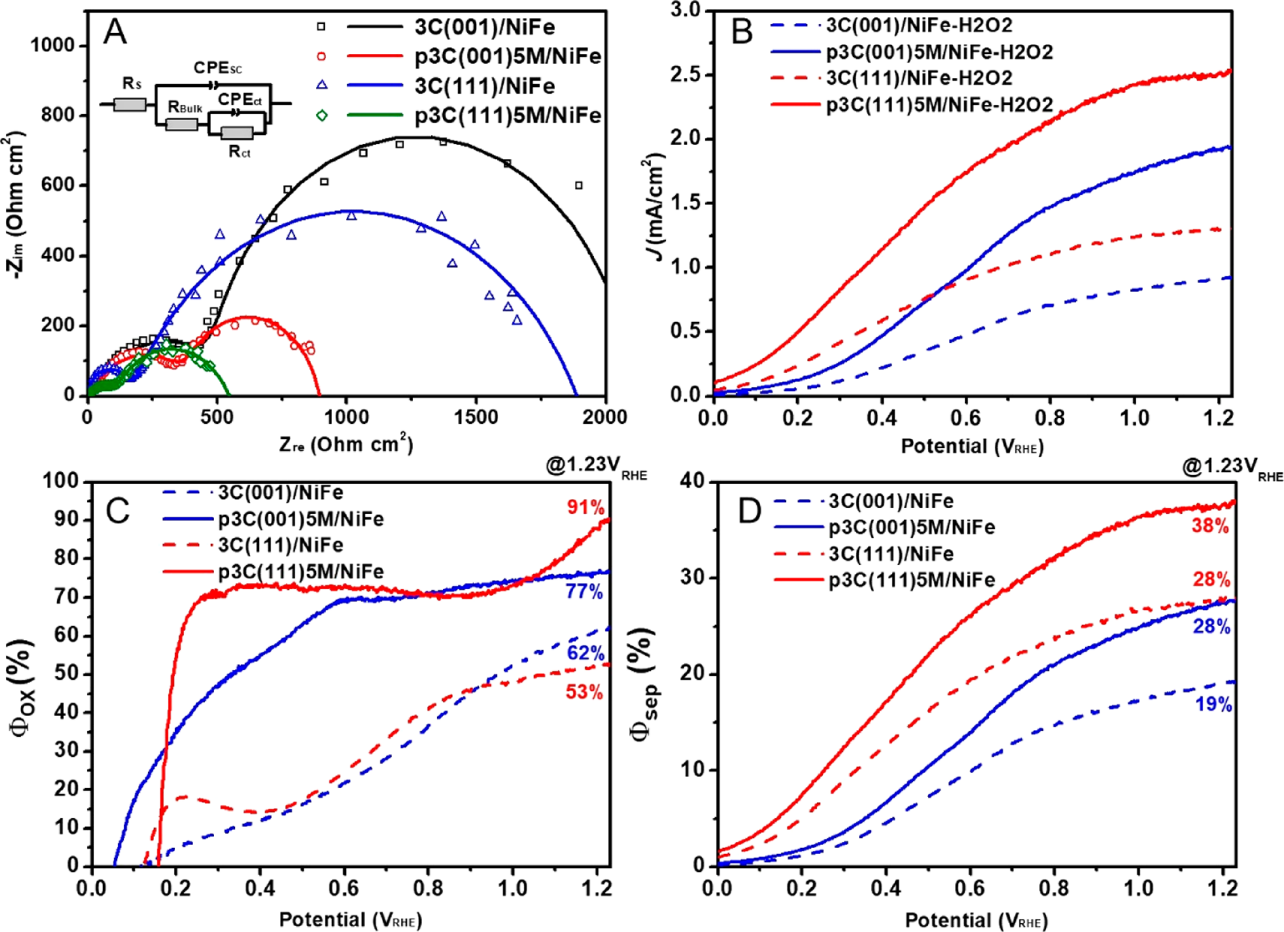
Effect of nanoporous structure of 3C-SiC on the charge transport properties, charge-separation efficiency (Φ_sep_), and charge-injection efficiency into electrolyte for oxidation reaction (Φ_ox_). (A) Nyquist plots of the 3C(001)/NiFe, p3C(001)5M/NiFe, 3C(111)/NiFe, and p3C(111)5M/NiFe photoanodes, measured from 1 to 10^5^ Hz at 1.23 V_RHE_ under AM1.5 100 mW cm^–2^ illumination. Panel A inset: equivalent circuit used for fitting the impedance data. (B) *J*–*V* curves of the 3C(001)/NiFe, p3C(001)5M/NiFe, 3C(111)/NiFe, and p3C(111)5M/NiFe photoanodes in 1.0 M NaOH electrolyte with 5% H_2_O_2_ as the hole scavenger under 1 sun illumination. Φ_ox_ (C) and Φ_sep_ (D) as a function of the potential V_RHE_ for the 3C(001)/NiFe, p3C(001)5M/NiFe, 3C(111)/NiFe, and p3C(111)5M/NiFe photoanodes.

To understand the significant improvement of the PEC water splitting performance of nanoporous 3C-SiC with respect to the planar counterparts, the electrochemical impedance spectroscopy (EIS) was measured at 1.23 V_RHE_ under AM1.5G, 100 mW cm^–2^ illumination in the frequency range of 1–10^5^ Hz. The Nyquist plots of all 3C(111)/NiFe, p3C(111)5M/NiFe, 3C(001)/NiFe, and p3C(001)5M/NiFe photoanodes exhibited two semicircles (Figure 6A), which were fitted by the equivalent circuit consisting of the series resistance (*R*_s_), the charge-transfer resistance (*R*_bulk_) and the capacitance (CPE_SC_) in the bulk of the photoanode, and the charge-transfer resistance from the photoanode to electrolyte (*R*_ct_) and the corresponding capacitance (CPE_ct_).^41^ The fitting results are listed in Table S3.

For the planar photoanodes, *R*_bulk_ in 3C(111)/NiFe is 3.4 times lower than that in 3C(001)/NiFe. This result can be explained by the higher crystalline quality of 3C-SiC(111), thus less charge recombinations in bulk compared to 3C-SiC(001), as confirmed by our previous work.^25,26^ We find that the XRD ω rocking curves of 3C-SiC(111) showed an average of the full width at half-maximum (fwhm) of 38 arcsec, which is 2.8 times smaller than that of 3C-SiC(001) measured under the same condition (fwhm = 105 arcsec for 3C-SiC(001)).^26,28^ In particular, from the low-temperature (2 K) photoluminescence measurement, we have observed the radiative emissions from up to four bound excitons in our high-quality 3C-SiC(111) material, indicating a suppressed nonradiative recombination and thus a longer carrier lifetime.^25^ The microwave photoconductivity decay measurements further confirmed that a long carrier lifetime of 8.2 μs was observed in our high-quality 3C-SiC(111), which is much longer than reported carrier lifetimes (a few to 120 ns) in 3C-SiC(001) grown on Si substrates.^25^

For the nanoporous photoanodes, both p3C(111)5M/NiFe and p3C(001)5M/NiFe significantly reduce *R*_bulk_ and *R*_ct_ compared to their planar counterparts (Table S3), which in turn explains their dramatic enhancement in the PEC performance. As expected, the nanoporous structure shortens the distances for charge transfer, provides a significantly enlarged surface area (see Tables 1 and 2), and increases the number of the catalytic sites for water oxidation, thus reducing both *R*_bulk_ and *R*_ct_.

The reflectance and transmittance spectra of the planar and nanoporous photoanodes were measured to extract their light-harvesting efficiency (LHE). As seen in Figures S11 and S12, both nanoporous 3C(111)*x*M/NiFe and 3C(001)*x*M/NiFe photoanodes exhibited a significantly reduced reflectance compared to their planar counterparts due to light trapping effect at the surface of the nanoporous structure. Planar 3C(111)/NiFe and 3C(001)/NiFe showed a high reflectance of over 20% at wavelengths less than 500 nm. With increasing the anodization time from 1 to 10 min, the resulting nanoporous photoanodes showed a gradual decrease of the reflectance, consistent with the increase of the surface area (Tables 1 and 2). With the anodization time ≥ 5 min, the nanoporous photoanodes showed a reflectance less than 5% in the wavelength range of <500 nm, which resulted in a high LHE of over 95% (Tables 1 and 2). This result reveals that nanoporous 3C-SiC photoanodes significantly reduce light reflectance, thus increasing the light absorption due to trapping effect.

To quantitatively demonstrate the effect of the nanoporous 3C-SiC structure on the improvement of charge-separation efficiency (Φ_sep_) and charge-injection efficiency into electrolyte for water oxidation (Φ_ox_), we measured the *J*–*V* curves of the photoanodes in 1 M NaOH with 5% H_2_O_2_ (Figure 6B). *J*_ph_ can be defined as *J*_ph_ = *J*_Abs_Φ_sep_Φ_ox_,^42^ where *J*_Abs_ is the photocurrent density converted from all absorbed photons by the photoanode at a 100% quantum efficiency. By integrating the solar AM1.5G spectrum with LHE, we obtained *J*_Abs_ for both nanoporous 3C(111)*x*M/NiFe and 3C(001)*x*M/NiFe photoanodes, which is also increasing with the increase of the anodization time (Tables 1 and 2). The improved *J*_Abs_ clearly evidence that the nanoporous structure enhances the light-harvesting efficiency. Since H_2_O_2_ is a hole scavenger enabling complete utilization of the photogenerated holes arrived at the electrode/electrolyte interface, namely, Φ_ox_(H_2_O_2_) = 100%, the photocurrent density measured with H_2_O_2_ in the electrolyte is given by *J*_H_2_O_2__ = *J*_Abs_Φ_sep_. Therefore, we can get Φ_ox_ = *J*_ph_/*J*_H_2_O_2__ and Φ_sep_ = *J*_H_2_O_2__/*J*_Abs_.

Figure 6C shows the calculated Φ_ox_ as a function of the potential for the planar and nanoporous photoanodes. Both the planar 3C(111)/NiFe and 3C(001)/NiFe showed very similar Φ_ox,_ which is expected due to the identical coating (in terms of morphology and thickness) of Ni:FeOOH cocatalyst. In contrast, the nanoporous p3C(111)5M/NiFe demonstrated a much steeper increase of Φ_ox_ than p3C(001)5M/NiFe and reached a plateau of ∼70% at a very low potential of ∼0.2 V_RHE_. This result is probably due to the presence of columnar pore structure in 3C(111) that facilitates the deposition of Ni:FeOOH in pores, thus increasing the number of the active catalytic sites. This explanation is supported by the larger surface area of nanoporous 3C(111) than that of nanoporous 3C(001), as seen in Tables 1 and 2. Moreover, it should be noted that p3C(111)5M/NiFe exhibited the highest Φ_ox_ of 91% at 1.23 V_RHE_, significantly outperforming its planar counterpart (Φ_ox_ = 53% at 1.23 V_RHE_ for 3C(111)/NiFe). Here, the results clearly reveal that compared to planar photoanodes, the nanoporous photoanodes significantly improve the charge-injection efficiency for water oxidation due to their large surface area that increases the number of the active catalytic sites.

Figure 6D shows the calculated Φ_sep_ curves of the planar and nanoporous photoanodes. Both the planar and nanoporous 3C-SiC(111) photoanodes showed higher charge-separation efficiency than the respective 3C-SiC(001) photoanodes. This result agrees very well with EIS results that smaller *R*_bulk_ was observed in both the planar and nanoporous 3C-SiC(111) photoanodes than those in the 3C-SiC(001) photoanodes (Table S3). A reason for these results may be that 3C-SiC(111) exhibited higher crystalline quality and longer carrier lifetime than 3C-SiC(001),^25,26^ as discussed in EIS results above.

As for the nanoporous photoanodes, both p3C(111)5M/NiFe and p3C(001)5M/NiFe exhibited a significant enhancement of Φ_sep_ with respect to their planar counterparts (Figure 6D). p3C(111)5M/NiFe showed the highest Φ_sep_ of 38%, and p3C(001)5M/NiFe exhibited the Φ_sep_ of 28% at 1.23 V_RHE_. Both are increased by a factor of ∼1.4 compared to their planar counterparts (Φ_sep_ = 28% for 3C(111)/NiFe and Φ_sep_ = 19% for 3C(111)/NiFe at 1.23 V_RHE_). In this work, we clearly demonstrate that nanoporous structure in 3C-SiC synergistically enhances light-harvesting efficiency, charge-separation efficiency, and charge-injection efficiency for water oxidation, thus evidently improving the overall PEC water splitting performance.

To further understand the improvements of nanoporous 3C-SiC photoanodes in PEC water splitting, we measured the incident photon-to-current efficiencies (IPCEs) of the 3C(111)/NiFe, p3C(111)5M/NiFe, 3C(001)/NiFe, and p3C(001)5M/NiFe photoanodes (Figure S13). For both 3C(111) and 3C(001), the nanoporous photoanodes exhibit higher IPCE than their planar counterparts. Compared to the IPCE of 20.1% for the planar 3C(111)/NiFe, the nanoporous p3C(111)5M/NiFe exhibited a higher IPCE of 28.3% at 410 nm. The improved IPCEs of nanoporous photoanodes agree very well with the enhancement of the photocurrent, light-harvesting, charge-separation, and charge-injection efficiencies.

## Conclusion

In summary, we have demonstrated a facile anodization method to fabricate nanoporous 3C-SiC photoanodes coupled with Ni:FeOOH cocatalyst that extraordinarily improve solar water splitting performance compared to their planar counterparts. The calculation results show that 3C-SiC exhibits a small value of (*L*_D_ + *W*_dep_) with respect to the large light penetration depth, which significantly limits the charge separation and results in a small photocurrent. We show that this drawback can be overcome by fabrication of nanoporous structure. The optimized nanoporous 3C-SiC photoanode achieved a high photocurrent density of 2.30 mA cm^–2^ at 1.23 V_RHE_ under 1 sun illumination, which is 3.3 times higher than that of its planar counterpart. To our knowledge, this PEC water splitting photocurrent is the highest value ever reported for 3C-SiC as a photoanode under AM1.5G 100 mW cm^–2^ illumination. The quantitative studies evidence that the nanoporous structure dramatically reduces light reflection, enlarges the electrochemically active surface area, and improves the charge-separation and -injection efficiencies, thus significantly enhancing the PEC water oxidation performance. Moreover, we find that the nanoporous 3C-SiC(111) shows a better PEC performance than the nanoporous 3C-SiC(001) due to its higher crystalline quality (thus higher Φ_sep_) and larger surface area (thus higher Φ_ox_). This work proposes a strategy for tailoring 3C-SiC for solar hydrogen production, which is also applicable for designing other indirect band gap semiconductors for solar energy conversion.

## Methods

### Materials

High-quality thick (∼1 mm) 3C-SiC(111) films were epitaxially grown on 4° off-axis 4H-SiC(0001) substrates by sublimation epitaxy growth.^26^ Then, freestanding, ∼300 μm thick 3C-SiC(111) films were obtained by polishing away the substrate and the interfacial layer. The commercial 3C-SiC(001) wafer (Hoya Corp.) was initially grown on Si(001) substrate and ∼260 μm thick freestanding 3C-SiC(001) were obtained after removal of the substrate. Prior to the preparation of photoanodes, 3C-SiC(111) and 3C-SiC(001) were chemically cleaned with acetone, ethanol, H_2_O:H_2_O_2_:NH_3_ (5:1:1), and H_2_O:H_2_O_2_:HCl (6:1:1) as well as HF (5%) solutions. Then, the 3C-SiC photoanodes were fabricated by the deposition of 200 nm thick Al Ohmic contacts on the backside of 3C-SiC, followed by an epoxy resin sealing of backside and edges so that only the surface was exposed to the electrolyte.

### Fabrication of Nanoporous 3C-SiC

The nanoporous 3C-SiC(111) and 3C-SiC(001) photoanodes were fabricated by an anodization method in a two-electrode cell under the same conditions. The planar 3C-SiC photoanode was connected to a 1 × 1 cm^2^ Pt cathode at 6 V (*versus* Pt) in a 5% HF solution under 410 nm LED illumination (∼50 mW cm^–2^). The nanoporous structure and depth in 3C-SiC(111) and 3C-SiC(001) were controlled by varying the anodization time from 1, 2, 5 to 10 min, respectively.

### Preparation of Ni:FeOOH Cocatalyst

Prior to PEC water splitting measurements, a Ni:FeOOH layer was deposited on 3C-SiC as the OER cocatalyst and the protection layer. For one-step deposition of Ni:FeOOH, a 25 mL precursor solution was prepared with 1 mM FeCl_3_ and 0.1 M NiCl_2_, considering that the solubility product constant of Ni(OH)_2_ (*K*_sp_ = 5.48 × 10^–16^) is much larger than that of FeOOH (*K*_sp_ = 2.79 × 10^–39^). In addition, 25 mL of urea (45 mM) was added to serve as the progressive OH^–^ releasing agent. Then, the 3C-SiC photoanodes were immersed in the solution, which was heated to 100 °C to evaporate half of the solution. Finally, the Ni:FeOOH-coated photoanodes were rinsed with deionized water and air-dried.

### Characterizations

The XRD measurements were performed using a Philips MRD with Cu Kα_1_ (λ = 1.54 Å). SEM and EDXS were measured by a LEO 1550 Gemini instrument. PEC measurements were carried out in a three-electrode cell by a potentiostat (Princeton Applied Research, VersaSTAT 3) in 1.0 M NaOH solution (pH = 13.6) under AM1.5G 100 mW cm^–2^ illumination from solar simulator (LOT-Quantum Design GmbH, calibrated by a standard Si photovoltaic cell). Prior to PEC measurements, the solution was deoxygenated by bubbling with high-purity (99.999%) Ar gas for over 30 min. The prepared 3C-SiC photoanode, 1 × 1 cm^2^ Pt plate, and Ag/AgCl (saturated KCl) were used as the working electrode, the counter electrode, and the reference electrode, respectively. The current density–potential measurements were performed at a scan rate of 30 mV s^–1^. The potential measured with respect to Ag/AgCl (*V*_Ag/AgCl_) was converted to the potential *versus* reversible hydrogen electrode (*V*_RHE_) using the following equation: *V*_RHE_ = *V*_Ag/AgCl_ + *E*_0_ + 0.059pH, where *E*_0_ is the potential of the Ag/AgCl reference electrode with respect to the standard hydrogen potential. The amounts of the evolved O_2_ and H_2_ gases were measured using a gas chromatograph (Agilent Technologies MicroGC490). The Faradaic efficiency was calculated by the ratio of the detected gas to the expected amount from photocurrent assuming 100% Faradaic efficiency.

## Supporting Information Available

Additional details on SEM images, elemental mapping, EDXS, XRD patterns, chopped *J*–*V* curves, cyclic voltammetry curves, reflectance spectra, transmittance spectra, light-harvesting efficiencies, and IPCE of the planar and nanoporous photoanodes; table showing a comparison of the photocurrent densities and ABPE of the reported 3C-SiC photoanodes (PDF)
