# Supplementary material for: Nanoporous Cubic Silicon Carbide Photoanodes for Enhanced Solar Water Splitting
Source: ACS Nano. 2021 Feb 19;15(3):5502–12. doi: 10.1021/acsnano.1c00256 (PMC8028336; doi:10.1021/acsnano.1c00256)
Supplement: Supplementary file 1 — nn1c00256_si_001.pdf [file nn1c00256_si_001.pdf]

Supporting Information

Nanoporous Cubic Silicon Carbide Photoanodes for Enhanced Solar Water Splitting

*Jing-Xin Jian, Valdas Jokubavicius, Mikael Syväjärvi, Rositsa Yakimova, and Jianwu Sun**

Department of Physics, Chemistry and Biology (IFM), Linköping University, SE-58183, Linköping, Sweden.

*Corresponding Author: Jianwu Sun, E-mail: jianwu.sun@liu.se

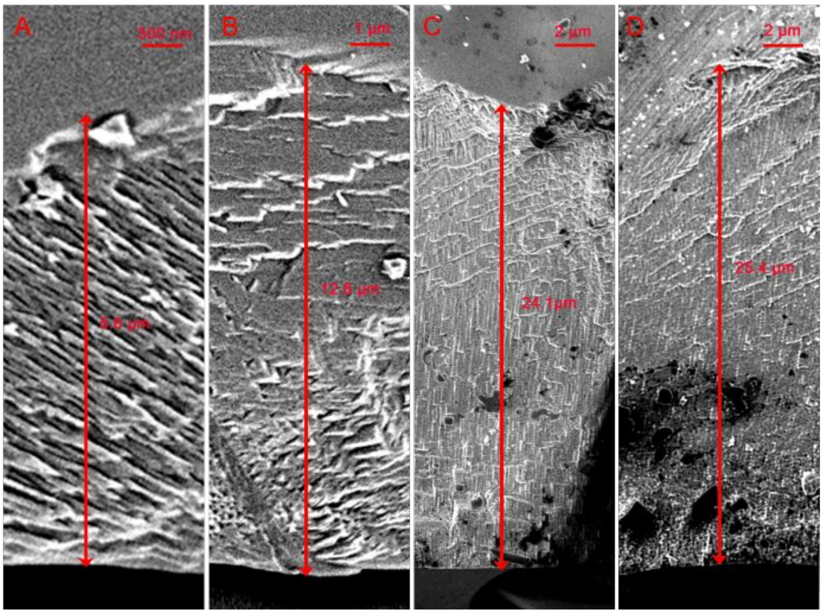

Figure S1. Cross-sectional SEM images of the nanoporous 3C-SiC(111) prepared by anodization for 1 min (A), 2 min (B), 5 min (C) and 10 min (D). The prepared samples are denoted as p3C(111)*x*M, where *x*M represents the anodization time of 1, 2, 5 and 10 minutes, respectively.

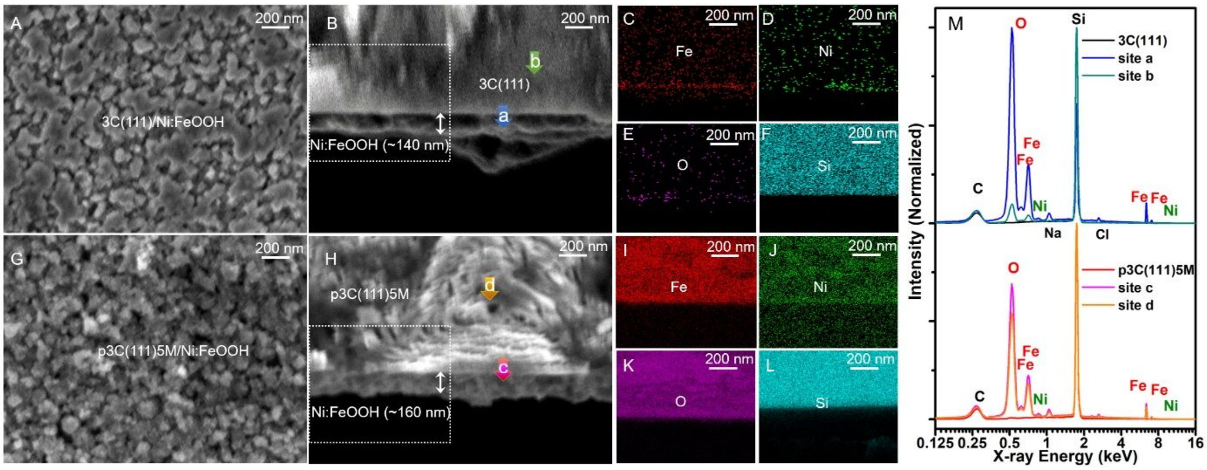

Figure S2. Top-view (A) and cross-sectional (B) SEM images of planar 3C(111)/NiFe. Elemental mapping of Fe (C), Ni (D), O (E) and Si (F) elements on the area indicated by the dotted box shown in the inset of (B). Top-view (G) and cross-sectional (H) SEM images of nanoporous p3C(111)5M/NiFe. Elemental mapping of Fe (I), Ni (J), O (K) and Si (L) elements on the area indicated by the dotted box shown in the inset of (H). (M) Energy-dispersive X-ray spectroscopy (EDXS) of 3C(111)/NiFe and p3C(111)5M/NiFe photoanodes measured at locations of a-d.

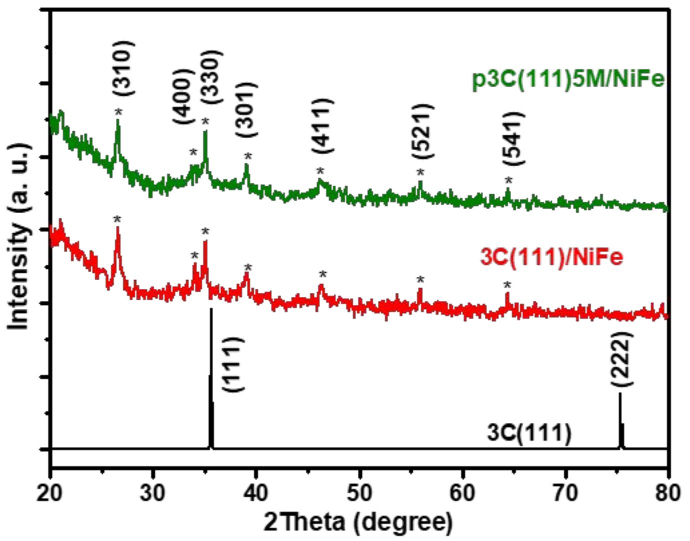

Figure S3. XRD patterns of planar 3C(111), 3C(111)/NiFe and nanoporous p3C(111)5M/NiFe photoanodes.

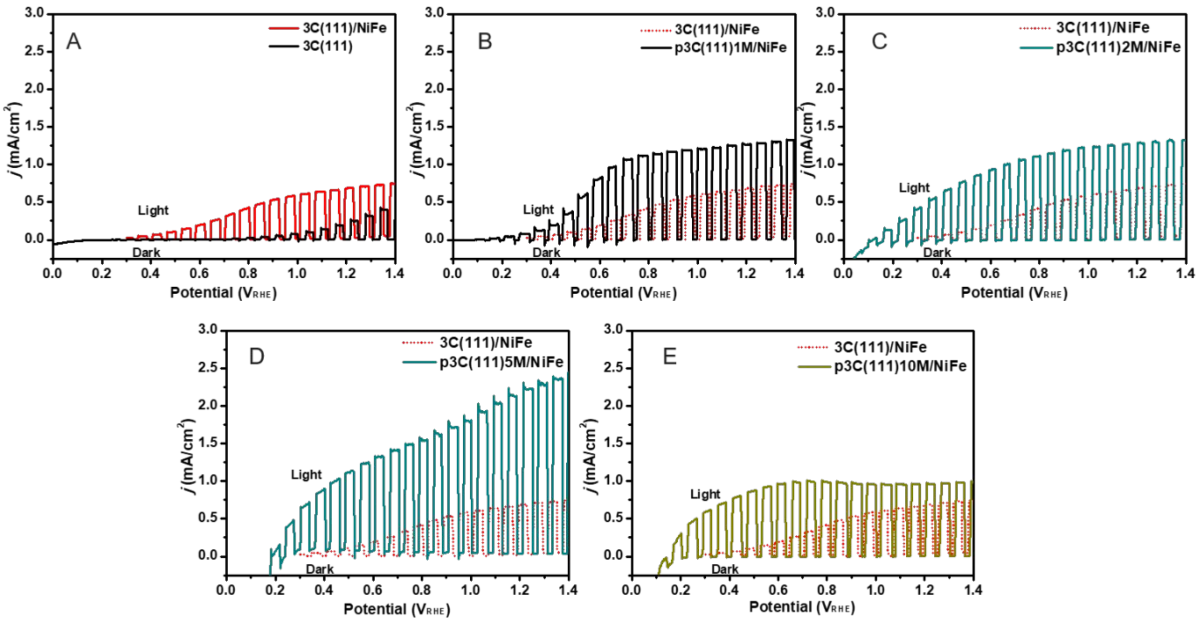

Figure S4. (A) *J-V* curves of planar 3C(111) and planar 3C(111)/NiFe photoanodes under chopped 1 sun illumination. The *J-V* curves of nanoporous photoanodes p3C(111)1M/NiFe (B), p3C(111)2M/NiFe (C), p3C(111)5M/NiFe (D) and p3C(111)10M/NiFe (E), compared to planar the 3C(111)/NiFe photoanode. All the measurements were carried out at scan rate of 30 mV s^{-1}, in 1.0 M NaOH electrolyte solution and under chopped AM1.5G 100 mW cm^{-2} illumination.

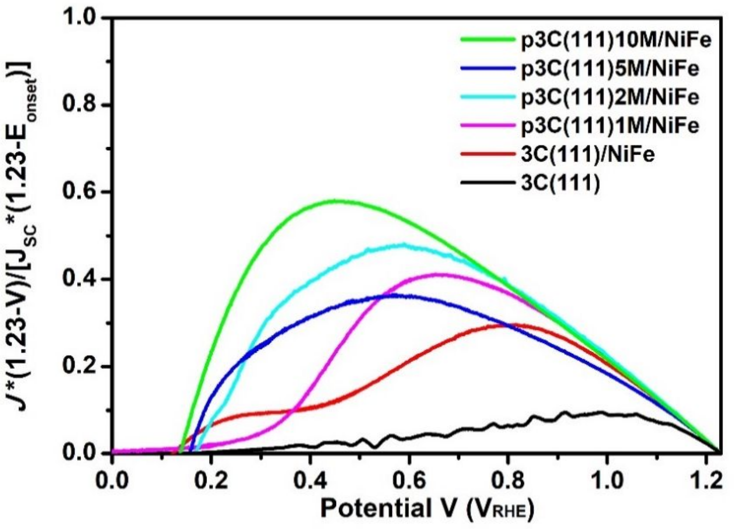

Figure S5. The plots of $J \times (1.23 - V)/[J_{sc} \times (1.23 - E_{onset})]$ *versus* V for the planar 3C(111), planar 3C(111)/NiFe and nanoporous p3C(111)*x*M/NiFe photoanodes (*x*M represents 1, 2, 5, 10 minutes). The maximum peak values of the plots give the fill factors of the *J*-*V* curves for the photoanodes, which are listed in Table 1.

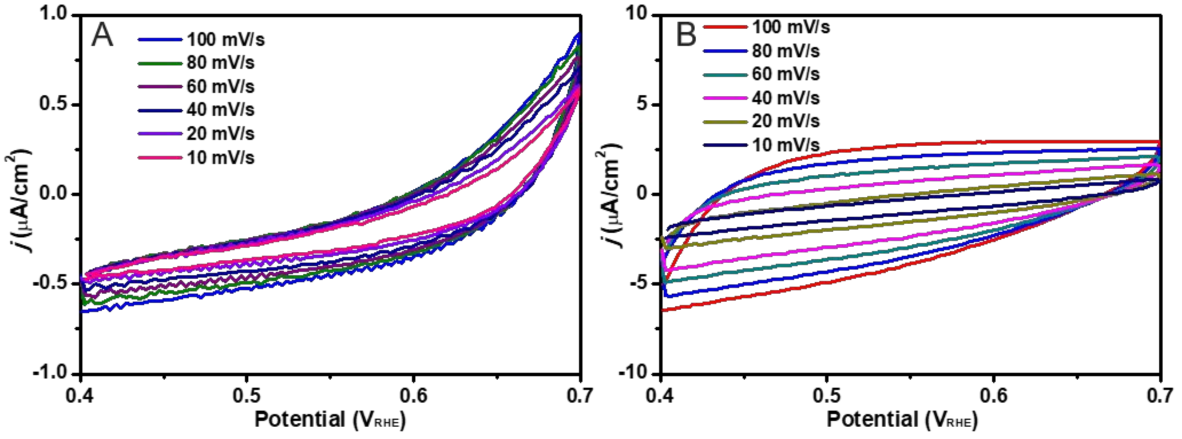

Figure S6. Cyclic voltammetry of the planar 3C(111)/NiFe (A) and the nanoporous p3C(111)5M/NiFe (B) photoanodes at different scan rates (*dV*/*dt*), in 1.0 M NaOH electrolyte solution in dark.

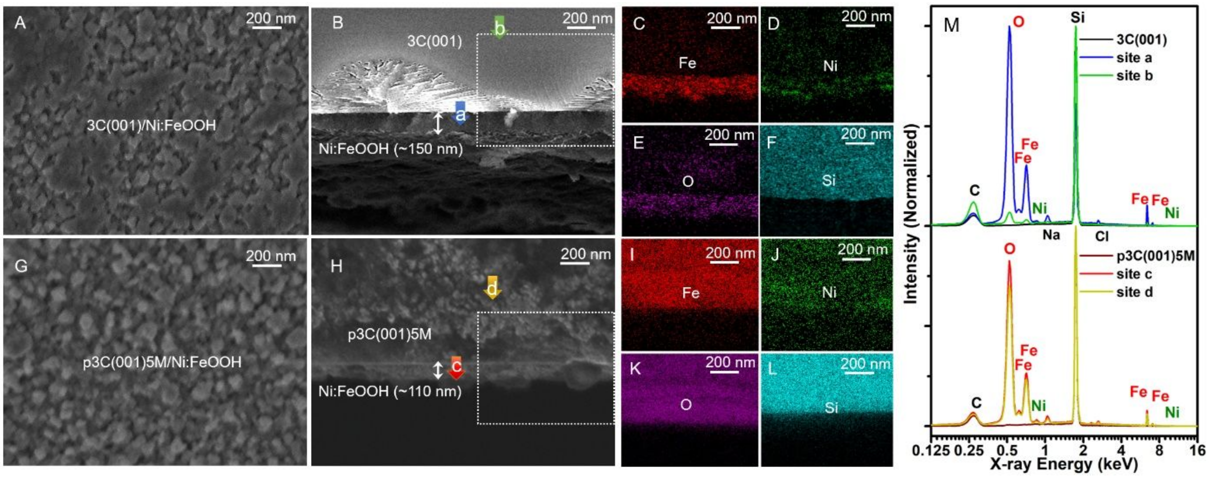

Figure S7. Top-view (A) and cross-sectional (B) SEM images of planar 3C(001)/NiFe. Elemental mapping of Fe (C), Ni (D), O (E) and Si (F) elements on the area indicated by the dotted box shown in the inset of (B). Top-view (G) and cross-sectional (H) SEM images of nanoporous p3C(001)5M/NiFe. Elemental mapping of Fe (I), Ni (J), O (K) and Si (L) elements on the area indicated by the dotted box shown in the inset of (H). (M) Energy-dispersive X-ray spectroscopy (EDXS) of 3C(001)/NiFe and p3C(001)5M/NiFe photoanodes measured at locations of a-d.

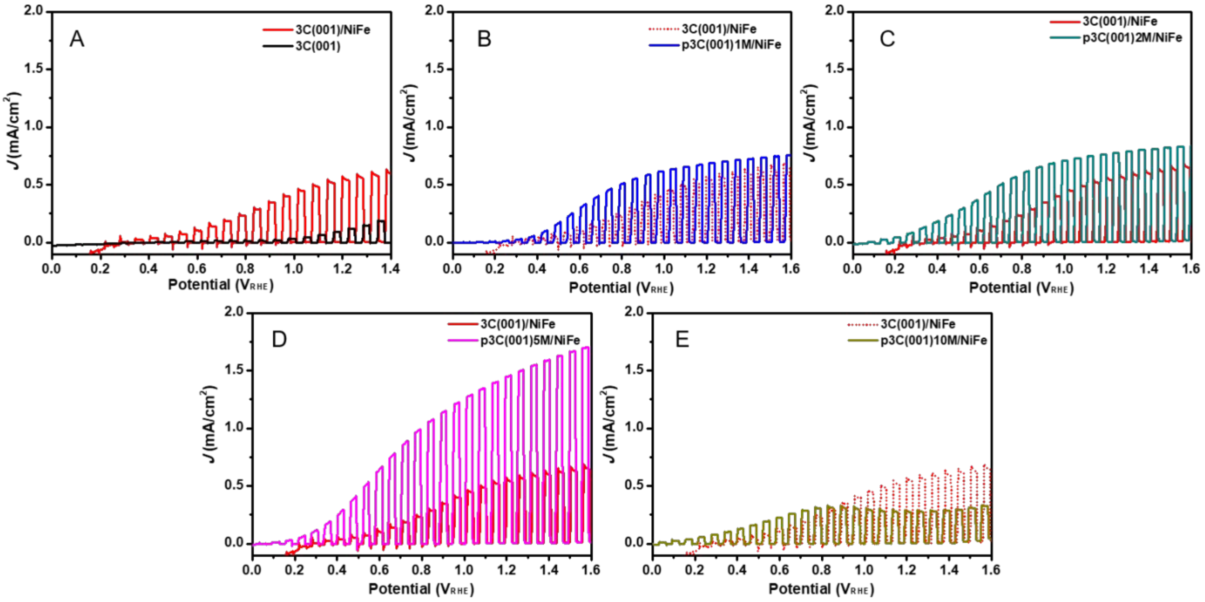

Figure S8. (A) *J-V* curves of the planar 3C(001) and planar 3C(001)/NiFe photoanodes. The *J-V* curves of nanoporous photoanodes, p3C(001)1M/NiFe (B), p3C(001)2M/NiFe (C), p3C(001)5M/NiFe (D) and p3C(001)10M/NiFe (E), compared to the planar 3C(001)/NiFe photoanode. All the measurements were carried out at scan rate of 30 mV s^{-1}, in 1.0 M NaOH electrolyte solution and under chopped AM1.5G 100 mW cm^{-2} illumination.

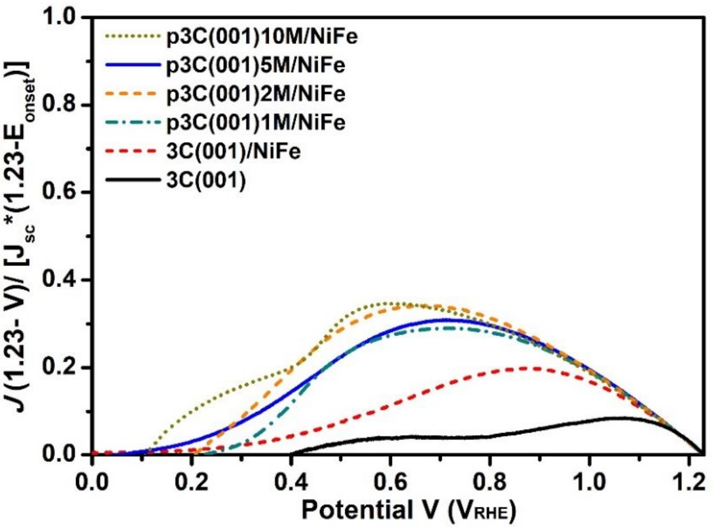

Figure S9. The plots of $J \times (1.23 - V)/[J_{sc} \times (1.23 - E_{onset})]$ *versus* V for the planar 3C(001), planar 3C(001)/NiFe and nanoporous 3C(001)xM/NiFe photoanodes, where xM represents the anodization time of 1, 2, 5 and 10 minutes. The maximum peak values of the plots give the fill factors of *J*-*V* curves for the photoanodes, which are listed in Table 2.

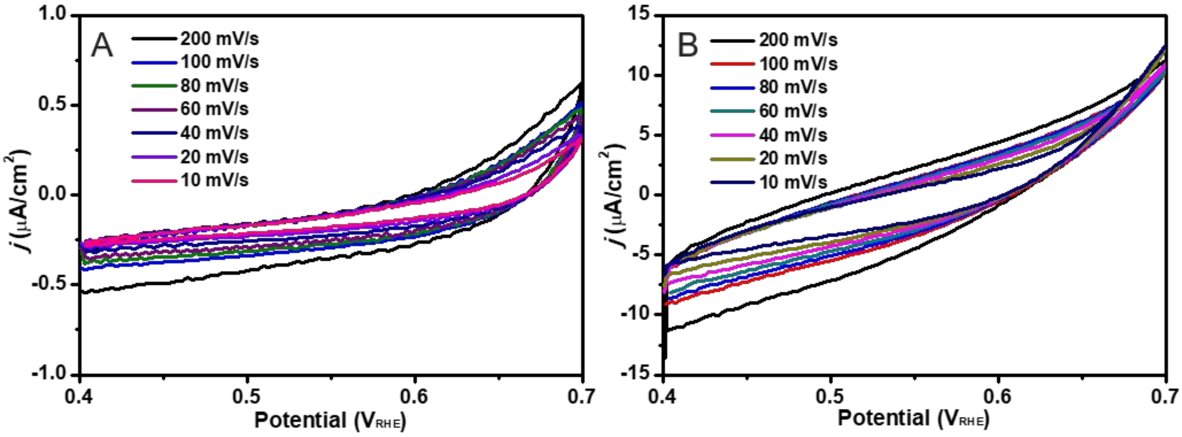

Figure S10. Cyclic voltammetry of the planar 3C(001)/NiFe (A) and nanoporous p3C(001)5M/NiFe (B) photoanodes at different scan rates (*dV*/*dt*), in 1.0 M NaOH electrolyte solution in dark.

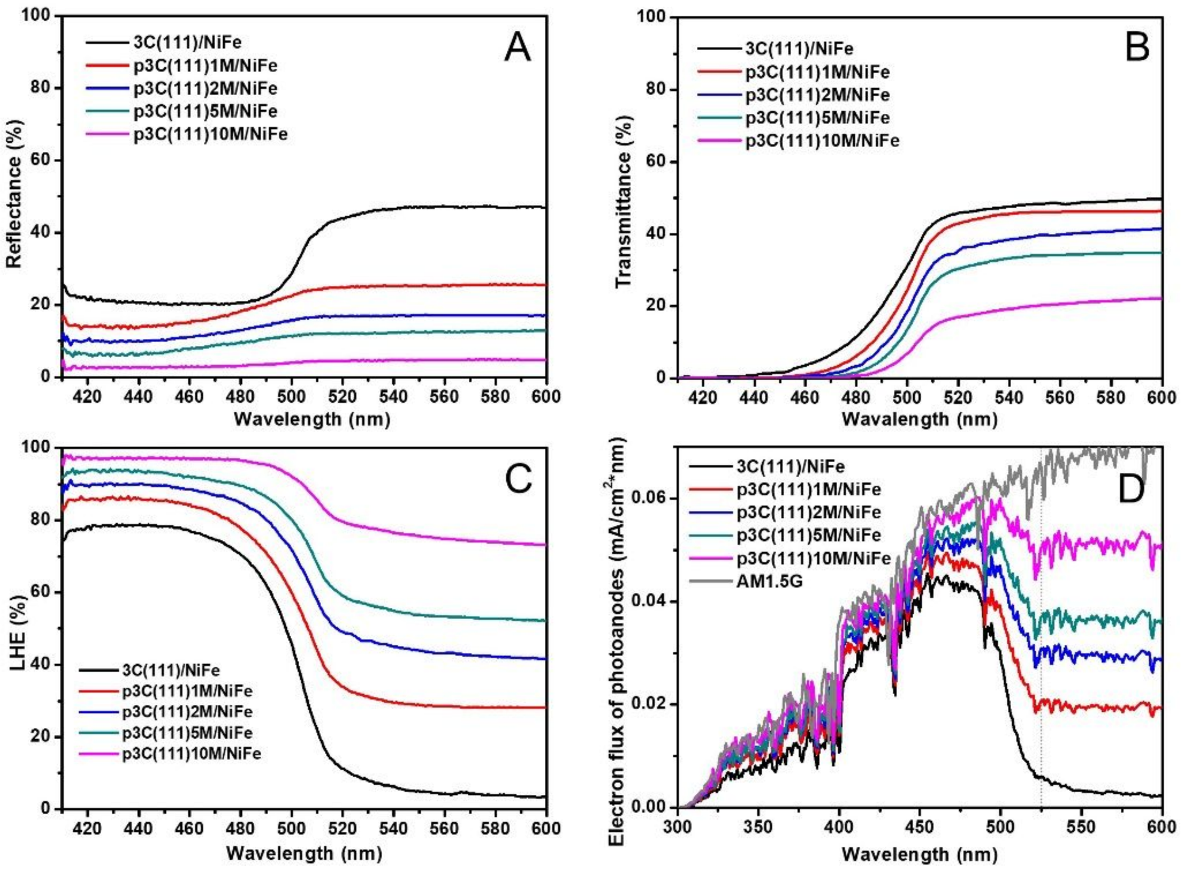

Figure S11. Reflectance spectra (A), transmittance spectra (B), light harvesting efficiency LHE (C), and the electron flux (D) of the planar 3C(111)/NiFe and the nanoporous p3C(111)*x*M/NiFe photoanodes, where *x*M represents the anodization time of 1, 2, 5 and 10 minutes. LHE of the planar and porous photoanodes calculated from the equation: LHE%=100 - transmittance - reflectance. The electron flux of the photoanodes was the product of the AM 1.5G electron flux and LHE. Photocurrent density (J_{abs}) at 100% internal quantum efficiency is calculated by integrating the electron flux at the photoanodes across 300-525 nm wavelength range. The bandgap of 3C-SiC is 2.36 eV (525 nm). J_{abs} values for photoanodes are listed in Table 1.

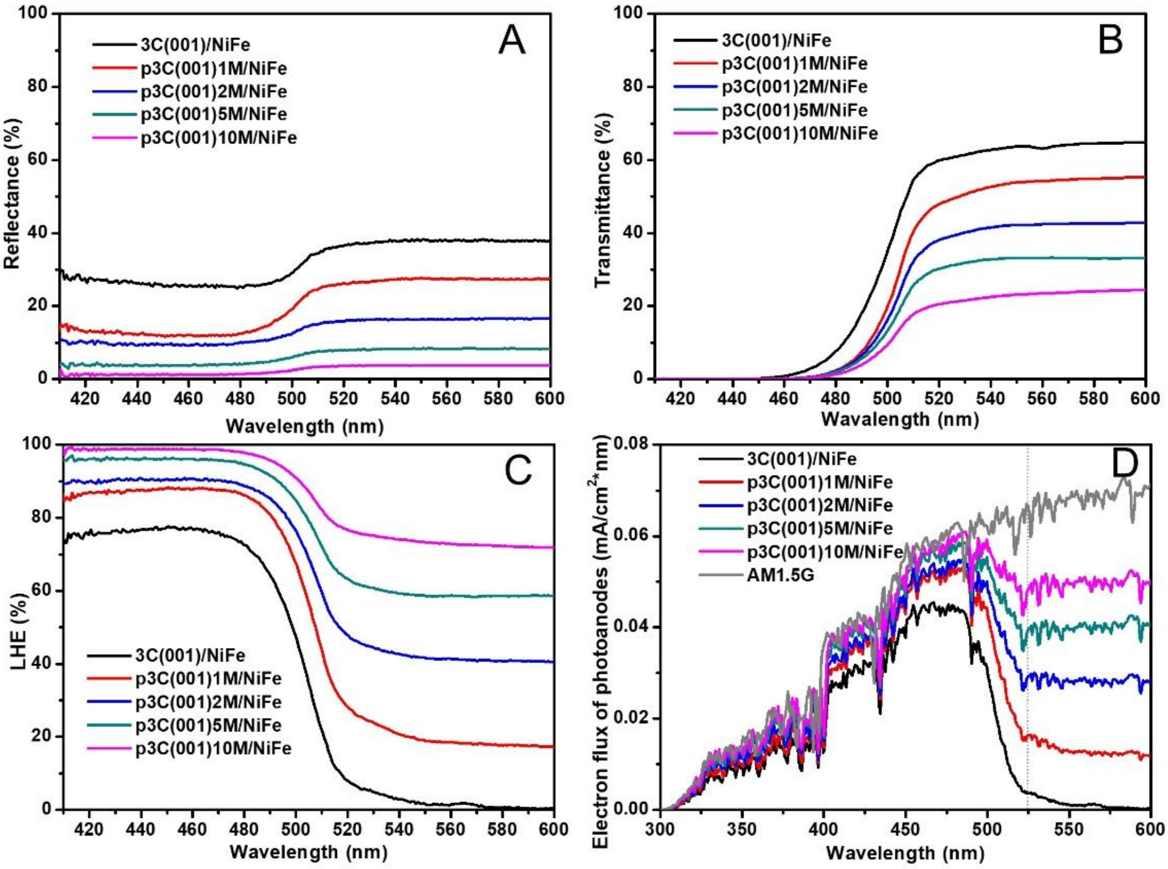

Figure S12. Reflectance spectra (A), transmittance spectra (B), light harvesting efficiency LHE (C), and the electron flux (D) of the planar 3C(001)/NiFe and the nanoporous p3C(001)*x*M/NiFe photoanodes, where *x*M represents the anodization time of 1, 2, 5 and 10 minutes. LHE of the planar and porous photoanodes calculated from the equation: LHE%=100 - transmittance - reflectance. The electron flux of the photoanodes was the product of the AM 1.5G electron flux and LHE. Photocurrent density (J_{abs}) at 100% internal quantum efficiency is calculated by integrating the electron flux at the photoanodes across 300-525 nm wavelength range. The bandgap of 3C-SiC is 2.36 eV (525 nm). J_{abs} values for photoanodes are listed in Table 2.

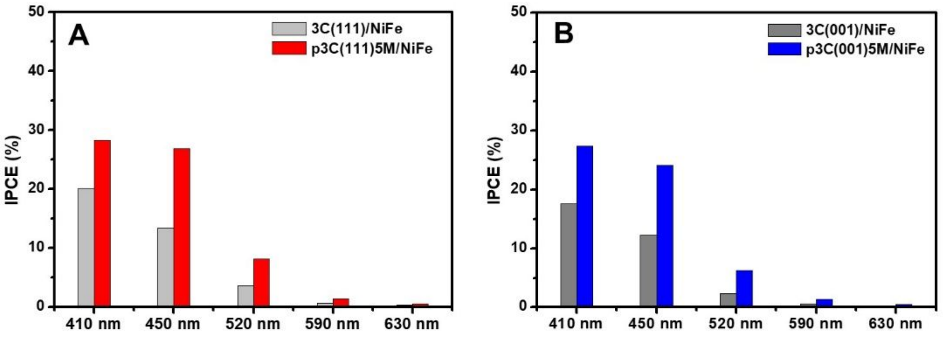

Figure S13. (A) Incident photon-to-current efficiencies (IPCE) of the planar 3C(111)/NiFe and nanoporous p3C(111)5M/NiFe. (B) IPCE of the planar 3C(001)/NiFe and nanoporous p3C(001)5M/NiFe. IPCE measurements were conducted at 1.23 V_{RHE} in 1.0 M NaOH under illuminations of different monochromatic LEDs with the spectral linewidth of 10 nm and a power density of 1 mW cm^{-2} on the sample surface.

Table S1. A comparison of the photocurrent densities and ABPE of 3C-SiC photoanodes for PEC water splitting reported recently.

**Photoanodes**	***J*** **@1.23** $\mathbf{V_{RHE}}$	**ABPE**	**PEC experimental conditions**	**Ref.**
3C-SiC	~0.11 mA cm^{-2}	--	400 mW cm^{-2} Xe light; 0.1 M Na_2SO_4	1
3C-SiC ([N]=1.0×$10^{16}$$cm^{-3}$)	0.20 mA cm^{-2} at 1.0 V *vs.* Ag/AgCl	--	994 mW cm^{-2} Solar-light lamp; 0.01M HCl	2
3C-SiC ([N]=3.6×$10^{18}$$cm^{-3}$)	0.13 mA cm^{-2} at 1.0 V *vs.* Ag/AgCl	--		
3C-SiC/Pt	2.03 mA cm^{-2} at 1.0 V *vs.* Ag/AgCl	--		
3C-SiC	0.70 mA cm^{-2} at 1.0 V *vs.* Ag/AgCl	--	Xe lamp, 100 mW cm^{-2}; 0.1 M $KHCO_3$,	3
3C-SiC(111)	0.12 mA cm^{-2}	--	100 mW cm^{-2} AM1.5G; 1.0 M NaOH	4
3C-SiC(111)/NiO	1.18 mA cm^{-2}	0.69%	100 mW cm^{-2} AM1.5G; 1.0 M NaOH	5
3C-SiC(111)/FeOOH	0.73 mA cm^{-2}	0.10%	100 mW cm^{-2} AM1.5G; 1.0 M NaOH	6
3C-SiC(111)/Ni:FeOOH	1.15 mA cm^{-2}	0.20%	100 mW cm^{-2} AM1.5G; 1.0 M NaOH	
3C-SiC(111)/monolayer-graphene	0.67 mA cm^{-2}	0.26%	100 mW cm^{-2} AM1.5G; 0.5 M $KHCO_3$	7
3C-SiC(111)/monolayer-graphene/FeOOH	1.14 mA cm^{-2}	0.61%	100 mW cm^{-2} AM1.5G; 0.5 M $KHCO_3$	
**Nanoporous p3C(001)5M/NiFe**	**1.50 mA** $\mathbf{cm^{-2}}$	**0.48%**	**100 mW** $\mathbf{cm^{-2}}$ **AM1.5G; 1.0 M NaOH**	**This work**
**Nanoporous p3C(111)5M/NiFe**	**2.31 mA** $\mathbf{cm^{-2}}$	**0.81%**	**100 mW** $\mathbf{cm^{-2}}$ **AM1.5G; 1.0 M NaOH**	

Table S2. The Faradaic efficiencies (**η_F**) of H_2 and O_2 for the nanoporous p3C(111)5M/NiFe and p3C(001)5M/NiFe photoanodes measured at 1.23 V_{RHE} under AM1.5G, 100 mW cm^{-2} illumination for 60 minutes.

The **η_F** of H_2 and O_2 was determined by the ratio of measured gas volume to the calculated gas volume from the photocurrent assuming 100% Faradaic efficiency. The figure below the table S2 shows the photocurrent density-time curves (A, C) and evolved volumes of H_2 and O_2 (B, D) for the nanoporous p3C(111)5M/NiFe and p3C(001)5M/NiFe photoanodes measured at 1.23 V_{RHE} under AM1.5G, 100 mW cm^{-2} illumination for 60 minutes. The 1×1 cm^2 Pt plate was used as the counter electrode (H_2 evolution). The evolved volumes of H_2 and O_2 were measured by gas chromatography (Micro-GC490, Agilent). The dotted lines show the calculated volumes of H_2 and O_2 from the photocurrent assuming 100% faradaic efficiency, respectively.

Photoanodes	$\eta_{F(O2)}$	$\eta_{F(H2)}$ *@ Pt electrode*
Nanoporous p3C(111)5M/NiFe	**75%**	91%
Nanoporous p3C(001)5M/NiFe	**73%**	94%

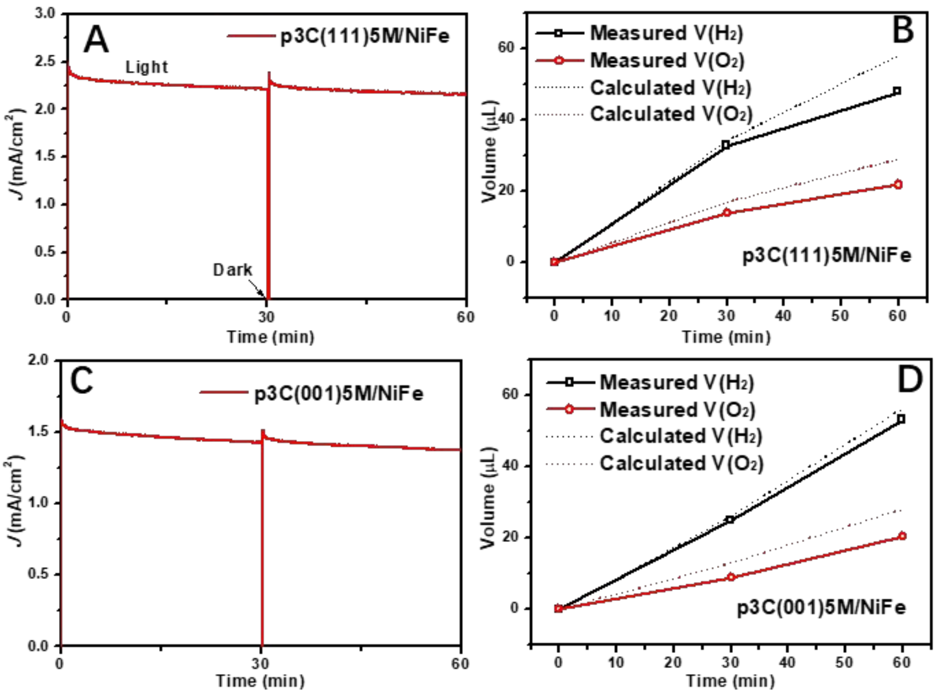

Table S3. Fitting results of the EIS data for planar 3C(001)/NiFe, 3C(111)/NiFe, and nanoporous p3C(001)5M/NiFe, p3C(001)5M/NiFe photoanodes.

Photoanodes	R_S (Ω*cm^2)	R_{bulk} (Ω*cm^2)	R_{ct} (Ω*cm^2)	CPE_{sc}-T	CPE_{sc}-P	CPE_{ct}-T	CPE_{ct}-P
Planar 3C(001)/NiFe	6.3	562	1568	1.26E-5	0.63	3.33E-5	0.97
p3C(001)5M/NiFe	5.1	411	477	9.38E-6	0.65	6.34E-5	0.91
Planar 3C(111)/NiFe	2.3	166	1772	4.98E-8	0.94	3.11E-5	0.70
p3C(111)5M/NiFe	1.1	125	419	3.19E-5	0.55	1.64E-4	0.72

References:

(1). Lauermann, I.; Memming, R.; Meissner, D., Electrochemical Properties of Silicon Carbide. *J. Electrochem. Soc.* **1997,** *144*, 73-80.

(2). Song, J. T.; Mashiko, H.; Kamiya, M.; Nakamine, Y.; Ohtomo, A.; Iwasaki, T.; Hatano, M., Improved Visible Light Driven Photoelectrochemical Properties of 3C-SiC Semiconductor with Pt Nanoparticles for Hydrogen Generation. *Appl. Phys. Lett.* **2013,** *103*, 213901.

(3). Song, J. T.; Iwasaki, T.; Hatano, M., Pt Co-Catalyst Effect on Photoelectrochemical Properties of 3C-SiC Photo-Anode. *Jpn. J. Appl. Phys.* **2014,** *53*, 05FZ04.

(4). Sun, J. W.; Jokubavicius, V.; Gao, L.; Booker, I.; Jansson, M.; Liu, X. Y.; Hofmann, J. P.; Hensen, E. J. M.; Linnarsson, M. K.; Wellmann, P. J.; Ramiro, I.; Martí, A.; Yakimova, R.; Syväjärvi, M., Solar Driven Energy Conversion Applications Based on 3C-SiC. *Mater. Sci. Forum* **2016,** *858*, 1028-1031.

(5). Jian, J. X.; Shi, Y. C.; Ekeroth, S.; Keraudy, J.; Syvajarvi, M.; Yakimova, R.; Helmersson, U.; Sun, J. W., A Nanostructured NiO/cubic SiC *p-n* Heterojunction Photoanode for Enhanced Solar Water Splitting. *J. Mater. Chem. A* **2019,** *7*, 4721-4728.

(6). Jian, J. X.; Shi, Y. C.; Syväjärvi, M.; Yakimova, R.; Sun, J. W., Cubic SiC Photoanode Coupling with Ni:FeOOH Oxygen-Evolution Cocatalyst for Sustainable Photoelectrochemical Water Oxidation. *Solar RRL* **2019,** *4*, 1900364.

(7). Li, H.; Shi, Y. C.; Shang, H.; Wang, W. M.; Lu, J.; Zakharov, A. A.; Hultman, L.; Uhrberg, R. I. G.; Syväjärvi, M.; Yakimova, R.; Zhang, L. Z.; Sun, J. W., Atomic-Scale Tuning of Graphene/Cubic SiC Schottky Junction for Stable Low-Bias Photoelectrochemical Solar-to-Fuel Conversion. *ACS Nano* **2020,** *14*, 4905-4915.
